# Source-Space Coverage Outweighs Receptor Placement in Sparse Urban Emission Inversion Under Matched and Moderately Misspecified Transport: A Mixed Point–Road Simulation Benchmark

**DOI:** 10.3390/s26216924

**Published:** 2026-11-01

**Authors:** Qiang Liu, Qingmin Hou, Qin Meng, Lunda Yan, Hanyang Zhang, Ziguang Jia

**Affiliations:** 1 School of Smart City, Chengdu Vocational & Technical College of Industry, Chengdu 610213, China; liuqiang@cdivtc.edu.cn (Q.L.); mengqin@cdivtc.edu.cn (Q.M.); yanlunda@cdivtc.edu.cn (L.Y.); 2 College of Computer Science, Sichuan University, Chengdu 610065, China; 3 School of Chemical Engineering, Ocean and Life Sciences, Dalian University of Technology, Panjin 124221, China

**Keywords:** urban air quality monitoring, sensor placement, low-cost sensor networks, emission inversion, source identification, source-inventory completeness, mixing height, optimal experimental design

## Abstract

Our work quantifies how much sensor placement affects emission-rate inversion for candidate urban facilities and road segments relative to other design decisions, given that monitoring networks are sited for concentration mapping. We built a paired benchmark covering 30 synthetic cities that places five design decisions on one error scale. Under matched transport, covering traffic in the source space changes normalized source error by 0.74, and dictionary fidelity and sensor calibration by about 0.68 each, against 0.13–0.15 across placement criteria; under an independent stability-dependent dispersion generator, the coverage effect shrinks to the order of the placement effect. Across five error types at matched severity, source identification degrades more slowly than emission rates, with similar patterns for every tested type. Unmodeled mixing height is among the most common errors (0.41–0.53), and an accurate external estimate can recover much of it. A truth-free residual flags severe model errors of every tested type (AUC 0.89–0.95). For seven Chengdu NO_2_ stations, poor out-of-sample skill, unstable support, and a large residual led us to withhold estimates. Where major inventory omissions or calibration errors exist, correcting them may reduce error more than switching among the placement criteria tested; identification capacity, rates, and residuals should be reported separately.

## 1. Introduction

Air quality management relies on emission inventories, which are used to design control measures, but urban inventories remain uncertain at the level of individual facilities and road links: sectors may be missing, fugitive and traffic emissions are poorly constrained, and reported rates may be outdated. Top-down emission estimation addresses this problem by inverting ambient observations through an atmospheric dispersion model to estimate the emission rates of candidate sources [1,2]. Such estimates could support the verification and updating of facility- and road-level inventories, which would in turn feed source attribution and air quality planning. The goal of our work is to quantify how much sensor placement affects the accuracy of emission-rate inversion for candidate urban facilities and road segments from a sparse monitoring network, relative to the other decisions made when the network and its inversion are set up, within a declared benchmark and perturbation ranges.

Emission-rate estimation must be distinguished from source attribution, a more common task in air quality management, which relates receptor concentrations to sources either backward, with receptor models [3,4] or forward, with dispersion or chemical-transport models [5,6], and in which emissions are inputs rather than unknowns. Emission-rate estimation reverses this direction: the rates are estimated from observations through a dispersion model [2,7]. Our work addresses only emission-rate estimation, referred to as *emission inversion* (Section 3.1), and makes no claim about source attribution.

Current emission inversion methods follow three main lines. Inverse dispersion with Gaussian, Lagrangian, or line-source models estimates the rates of one or a few known sources from nearby, purpose-placed samplers [7,8,9]. Source-term estimation for accidental releases infers the location and rate of a single unknown source, often with sensor placement optimized for that purpose [2,10,11]. City-scale network inversions estimate gridded or sectoral fluxes, often as scaling factors on a prior inventory [12,13], and satellite NO_2_ retrievals are increasingly used to estimate city and large-facility emissions top-down [14,15,16]. Each line has a limitation when routine urban emissions from many facilities and roads are to be estimated with an existing sparse network. Single-source methods do not address many simultaneous emitters of different geometries. Gridded and satellite-based estimates, at the resolutions used in the cited studies, do not separate individual facilities from nearby road segments, and inversions that scale a prior inventory can correct a sector only if the prior represents it. Most importantly, studies that optimize sensor placement for emission estimation [11,17,18] typically compare placement criteria against each other while holding the source description, transport model, and sensor quality fixed. Meanwhile, regulatory and low-cost networks are sited for compliance or concentration mapping [19,20,21], not for inversion.

Consequently, it remains unclear whether improving sensor placement is the decision that most limits urban emission inversion, or whether other decisions made when a network and its inversion are set up have larger effects. These include whether the candidate source list covers every emitting sector, whether candidate locations are correct, and how well sensors are calibrated. Without such a comparison, it is difficult to judge whether additional or relocated monitors, a more complete source inventory, or better sensor calibration would change inversion error most under given conditions.

To address this goal, we ask: *how strong is the effect of sensor placement on the accuracy of urban emission inversion, compared with the effects of source-inventory completeness, candidate-location accuracy, transport and boundary-layer fidelity, and sensor calibration?* To answer it, we pursue four objectives:

(O1) To measure, on a common error scale and for the same simulated cities, sources, and weather, the effect of each of these design decisions on emission-rate error and on source-identification accuracy;
(O2) To test whether these relative effects, and in particular two pairwise comparisons (dictionaries with and without road atoms; line atoms versus densified point atoms), are robust to transport models that are different from the one used for inversion and to narrower perturbation ranges;
(O3) To determine whether source identification and emission rates degrade differently under model error, and hence whether they should be reported separately;
(O4) To provide a truth-free diagnostic of model inadequacy and to examine, on a real monitoring subset, whether the available evidence supports reporting an emission estimate.

Our work combines established components (a dispersion-based source–receptor operator, nonnegative regularized inversion, and optimal-design placement) into a paired simulation benchmark over 30 synthetic cities, supplemented by controlled-release data and a real station subset in Chengdu. It does not propose a new estimator. In brief, the results indicate that, within the declared ranges, inventory completeness and sensor calibration can matter more than the choice among placement criteria, with effect magnitudes that depend on transport fidelity and on the ranges assumed. All quantitative results are simulation-based unless stated otherwise, and the conclusions are conditional on the declared framework and perturbation ranges (Section 8).

**Figure 1:**
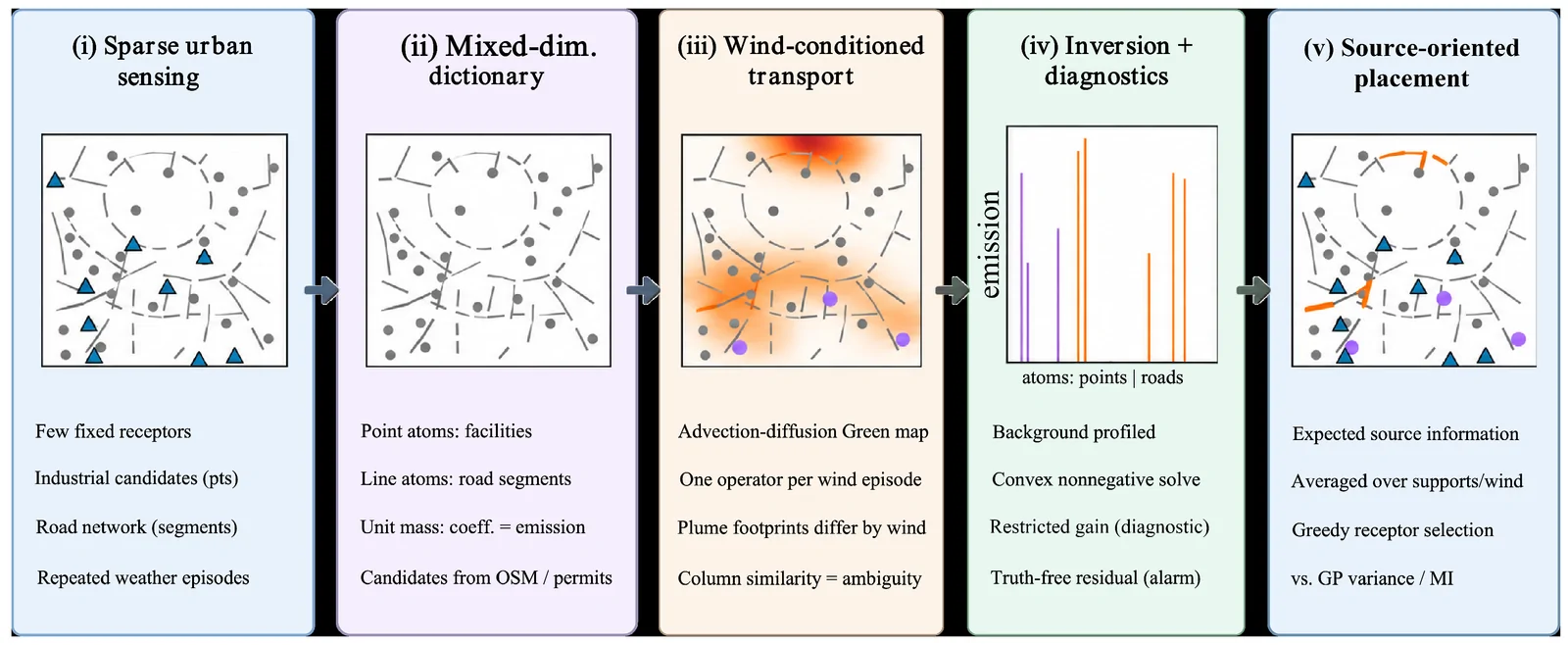
Conceptual workflow. Sparse urban receptors observe a city with industrial point sources and road traffic; candidate facilities and road segments form a mixed point–line source dictionary; a wind-conditioned dispersion operator links candidate emissions to receptor observations; a nonnegative inversion estimates which candidates emit and at what rate, a truth-free residual flags model inadequacy, and a placement step selects receptors. Blue triangles, receptors; grey dots, candidate point facilities; grey line segments, candidate road segments; orange shading, wind-dependent plume footprints; purple and orange markers/bars, active point and road sources, respectively. Details are given in Section 3, Section 4 and Section 5.

Section 2 reviews related work. Section 3 defines terminology, the transport model, and the source space; Section 4 and Section 5 give the identifiability analysis and the inversion and placement methods. Section 6 describes the experiments, Section 7 the results, and Section 8 the discussion and limitations. Figure 1 summarizes the workflow.

## 2. Related Work

### 2.1. Field Reconstruction from Sparse Urban Sensors

Urban field inference combines sparse stations with land use, meteorology, traffic, or graph structure: U-Air couples spatial and temporal classifiers with urban covariates [22], and graph or neural kriging methods reconstruct unseen nodes [23,24,25]. Their target is concentration at unobserved locations; we retain field error as a metric but estimate a different latent variable, source support and intensity. Mobile monitoring [26] and satellite NO_2_ retrievals [14,15] can support construction of the candidate dictionary, whose coverage has the largest effect in our comparisons. Station-siting recommendation from urban big data [19] and multi-type placement in Gaussian spatial fields [21] optimize concentration coverage. GP mutual information is the classical near-optimal placement rule for field reconstruction [20]; because variance maximization pushes sensors toward domain boundaries whereas mutual information retains interior coverage, we include both criteria to assess how the mapping–inversion contrast depends on the field-oriented baseline.

### 2.2. Low-Cost Sensor Networks and Data Quality

Low-cost nodes exhibit large between-unit variability and month-scale drift [27,28,29,30]; calibration models degrade and require periodic co-location [31,32,33], motivating in-situ and network-based drift correction [34,35], nationwide corrections [36], fusion into exposure models [37], and calibration-first network design [38]. This literature evaluates data quality against *concentration* accuracy; RQ3 asks the corresponding question for emission inversion and finds that persistent multiplicative drift, which repeated weather cannot average away, dominates every placement decision we test.

### 2.3. Atmospheric and PDE-Constrained Source Inversion

Atmospheric inversions construct source–receptor matrices with Eulerian or Lagrangian transport [1,39]; Bayesian and building-resolving urban source determination [10,40] and multi-point Gaussian plume inversion [8] are established. City-network CO_2_ studies estimate sectoral emissions including highways and industrial sources [12,13,41,42], but aggregate emissions over prescribed spatial patterns rather than resolving candidate-level point and road atoms; receptor-model apportionment resolves chemical source *types*, not located emitters [4,43,44]. Because transport error can dominate observation noise [45], we retain a mismatch term in the stability result and run out-of-model simulations. Inverse-source theory for point sources and sparse measure-valued sources [46,47,48] underlies our identifiability analysis, which is scoped to finite candidate measures with nuisance-profiled stability.

### 2.4. Optimal Experimental Design

Sensor placement for inverse problems is formulated through A-, D-, or goal-oriented optimal design [17,18,49,50], coupled with source identification for advection–diffusion systems [11,51], and evaluated for single accidental releases in urban neighbourhoods with Bayesian and CFD forward models [52,53,54]. We address many simultaneous routine emitters, take expectation over sparse mixed-dimensional supports and weather, and state when nuisance profiling preserves or destroys submodularity.

## 3. Problem Formulation

### 3.1. Terminology and Positioning

*Emission inversion* denotes estimating the nonnegative emission rates θ of a *predefined candidate source dictionary* (a finite list of candidate facilities and road segments) from receptor observations through a dispersion model. It has two components, scored separately: *source identification* (support identification), deciding which candidates have $\theta_{g}>0$, scored by support AUPRC; and *emission-rate estimation*, recovering the magnitudes, scored by normalized source error. *Source estimate* refers to the output of both. Emission inversion differs from *source attribution* (source apportionment), which does not estimate emission rates. Backward attribution uses receptor models such as positive matrix factorization or chemical mass balance on speciated measurements [3,4]. Forward approaches comprise two distinct quantities [5,6]: source tagging quantifies source *contributions* to concentrations, whereas emission-perturbation (sensitivity) simulations quantify concentration *responses* to emission changes. These coincide under linear concentration–emission relationships but need not coincide under nonlinear chemistry. The transport model used here is linear in emissions (a primary, non-reactive tracer), so within this framework a source’s contribution and its emission-perturbation response are identical; this equivalence does not extend to secondary or chemically reactive pollutants. Neither receptor-model apportionment nor regulatory assignment of responsibility is performed here, and every estimate is conditional on the declared dictionary and transport model. *Source-space completeness* (or *coverage*) denotes whether every emitting sector and location is represented by at least one dictionary column. It is the inversion-side counterpart of *source-inventory completeness*: an inventory that omits a sector (e.g., traffic, fugitive, or area emissions) yields a dictionary with no columns for it, and the inversion must then ignore or misassign that mass. We use *coverage* for whether a whole sector (traffic) is present, and *dictionary fidelity* for location or composition errors of sources within or beyond the declared sectors (Section 7.2).

The components are standard: source–receptor matrices [1,39], regularized sparse or nonnegative inversion [8,10,47], line-source dispersion [9], and Bayesian optimal design [17,18]. Our work departs from previous work in three respects, none of which is a new estimator: it solves for individual candidate facilities and road segments represented as point and line measures, so that inventory completeness becomes a testable design variable; it varies source space, dictionary fidelity, sensor grade, wind excitation, and placement on one paired protocol; and it couples each estimate to a truth-free residual, same-network surrogate tests, and out-of-sample checks, so that the pipeline can decline to report an estimate. We refer to the pipeline as MIST (Mixed-dimensional Inversion of Sources under Transport); its placement variant is introduced in Section 5.2. Industrial candidates are point measures and traffic emissions are supported on road segments, consistent with line-source models such as RLINE [9].

### 3.2. Transport and Observation Model

Let $\Omega \subset R^{2}$ be an urban domain. Meteorological episode r∈{1,…,R} obeys

(1) $$\partial_{t}u_{r}-\nabla \;\cdot \left(K_{r}\nabla u_{r}\right)+b_{r}\;\cdot \nabla u_{r}+c_{r}u_{r}=S_{\theta }\;\mathrm{in}\;\Omega \times \left(0,T\right),$$

with well-posed initial and boundary conditions. Denote the linear source-to-state solution operator by $G_{r}$. A receptor applies a bounded space–time averaging functional $H_{ri}$, giving

(2) $$y_{ri}=H_{ri}G_{r}S_{\theta }+B_{ri}\eta_{r}+\varepsilon_{ri}.$$

Here $B_{r}\eta_{r}$ represents episode-level background offset and gradient terms, and ε∼N(0,Σ). Unknown transport coefficients can be added to η after local linearization; source–transport confounding then appears explicitly in the effective Jacobian.

### 3.3. Mixed-Dimensional Source Space

Let $p_{j}$ be an industrial candidate and $\gamma_{e}:\left[0,L_{e}\right]\to \Omega$ a road segment. For a nonnegative unit-mass footprint kernel $\kappa_{\sigma }$, define

(3) $$\phi_{j}^{\mathrm{pt}}\left(x\right)=\kappa_{\sigma }\left(x-p_{j}\right),\;\phi_{e}^{\ln}\left(x\right)=\frac{1}{L_{e}}\int_{0}^{L_{e}}\kappa_{\sigma }\left(x-\gamma_{e}\left(s\right)\right)\;ds.$$

Both atoms integrate to one, so their coefficients represent total emissions. With low-dimensional temporal bases $\psi_{h}\left(t\right)$,

(4) $$S_{\theta }\left(x,t\right)=\sum_{g=1}^{P}\sum_{h=1}^{q}\theta_{gh}\phi_{g}\left(x\right)\psi_{h}\left(t\right),\;\theta_{gh}\geq 0\;\left(q=1\;\mathrm{throughout}\;\mathrm{the}\;\mathrm{experiments}\right).$$

The spatial support is group sparse across time bases. The present experiments use q=1, meaning that source intensity is shared within a short campaign while weather changes. This assumption supplies repeated physical experiments; letting every episode have unrelated emissions would add as many unknowns as observations. The assumption is nevertheless restrictive and its effect should be stated explicitly. If true emissions vary across episodes as $\theta_{r}=\bar{\theta }+\delta_{r}$ while the inverse imposes a single θ, the unmodeled part $A_{r}\delta_{r}$ enters the mismatch term ρ of (5) and is amplified by $1/\alpha_{C,B}$ in (9). Two cases differ in severity. Variation that is independent of meteorology averages towards zero in the pooled fit and mainly inflates variance, so the estimate approaches the episode-mean emission $\bar{\theta }$. Variation that is *correlated* with meteorology—for example, traffic peaking in the stable, low-wind morning and evening hours, or heating emissions in cold, stagnant episodes—is not averaged out: the inversion cannot separate an emission increase from the change of transport that accompanies it, so the bias is systematic and falls mainly on the road atoms and their nearest neighbours. Section 7.7 quantifies the second case with a two-peak diurnal traffic profile that the q=1 inverse does not represent: source error rises from 0.087 to 0.29–0.35 at realistic amplitude, whereas support AUPRC remains above 0.93. The constant-intensity results should therefore be read as applying to campaigns stratified by time of day (or season), and the reported magnitudes as episode-averaged rates. Equation (4) already admits q>1 (e.g., a periodic diurnal basis or separate weekday/weekend coefficients) at the cost of *q* times more unknowns per atom, which reduces the number of episodes available per unknown; evaluating this trade-off is left to future work.

Stacking episodes yields

(5) $$y=A\theta +B\eta +\varepsilon +\rho ,\;A_{\left(r,i),(g,h\right)}=H_{ri}G_{r}\left(\phi_{g}\psi_{h}\right),$$

where ρ denotes transport, discretization, and dictionary mismatch. Given θ and transport, $u_{r}=G_{r}S_{\theta }$ is a derived state, not an independently identifiable unknown.

## 4. Identifiability

### 4.1. Why Structure Is Necessary

Unrestricted source recovery from finitely many observations is non-identifiable because a linear map from an infinite-dimensional source class to $R^{M}$ has an infinite-dimensional kernel, and nonnegativity does not remove the ambiguity (Supplementary Section S1 gives the argument using M+1 disjoint bumps). The point–road dictionary therefore changes the problem from arbitrary function recovery to inference on a finite union of low-dimensional cones, and the analysis below concerns the stable solvability of this restricted problem.

### 4.2. Stable Identifiability After Profiling Nuisance

Whiten the observations: $\tilde{A}=\Sigma^{-1/2}A$ and $\tilde{B}=\Sigma^{-1/2}B$. Let

(6) $$P_{B}^{⊥}=I-\tilde{B}{\tilde{B}}^{†}.$$

For a feasible parameter set C, define its difference set D(C)=C−C and the nuisance-profiled restricted gain

(7) $$\alpha_{C,B}=\underset{\delta \in D(C)\setminus \{0\}}{\inf}\frac{{∥P_{B}^{⊥}\tilde{A}\delta ∥}_{2}}{{∥\delta ∥}_{2}}.$$

**Proposition 1**  (Restricted identifiability and stability)**.** *The source is globally identifiable on C modulo nuisance if and only if $\ker\left(P_{B}^{⊥}\tilde{A}\right)\cap D\left(C\right)=\left\{0\right\}$. If $\alpha_{C,B}>0$ and*

(8) $$\hat{\theta }\in \underset{\theta \in C}{\arg\;\min}\min_{\eta }{∥\Sigma^{-1/2}\left(y-A\theta -B\eta \right)∥}_{2}^{2},$$

*then*

(9) $${∥\hat{\theta }-\theta^{*}∥}_{2}\leq \frac{2{∥P_{B}^{⊥}\Sigma^{-1/2}\left(\varepsilon +\rho \right)∥}_{2}}{\alpha_{C,B}}.$$

**Proof.**  See Supplementary Material, Section S1. □

Proposition 1 bounds only the unregularized constrained-least-squares estimator, not the regularized estimator of Section 5, whose penalty introduces bias.

For a known support *T*, the gain is $\sigma_{\min}\left(P_{B}^{⊥}{\tilde{A}}_{T}\right)$. Uniform recovery of signed *s*-sparse parameters is guaranteed by a positive 2s-restricted gain; the exact nonnegative condition can be weaker because its difference set is smaller. Equation (9) also shows why a wind error ΔA is dangerous: it contributes $\rho_{\mathrm{wind}}=\Delta A\theta^{*}$ and is amplified by $1/\alpha_{C,B}$.

The classical coherence condition $\sigma_{\min}^{2}\left(A_{T}\right)\geq 1-\left(|T|-1\right)\mu$ (Supplementary Section S3) is vacuous here—our operators have μ≈0.96–0.998 because nearby road atoms under smooth transport are intrinsically near-collinear—and μ is used only as a relative diagnostic of whether weather diversity reduces column similarity.

### 4.3. Restricted Gain on the Nonnegative Feasible Difference Set

The interpretation of Proposition 1 depends on the feasible difference set used to evaluate the restricted gain. If D(C) is taken to be the set of all 2s-sparse *signed* vectors—the conventional choice, which is what the coherence literature bounds—then on our operators the sampled gain is $\alpha_{C,B}=0.061$ ([0.050,0.073]), and (9) predicts errors far larger than those we measure. Emissions are nonnegative, so the feasible set is a cone and the relevant difference set is $D\left(C\right)=\left\{\theta_{1}-\theta_{2}:\theta_{1},\theta_{2}\geq 0,\;\mathrm{supp}\theta_{i}\in S\right\}$, which is strictly smaller than the signed set. A natural conjecture, stated in the submitted version, is that the cone excludes the sign-alternating combinations of near-collinear road atoms and therefore raises the gain substantially. A deeper analysis, performed in response to review, shows that this is not the case for the worst-case gain, and we report the corrected result here.

*How the cone gain was computed and why its Monte Carlo minimum does not converge.* The infimum over a difference of two cones is a nonconvex problem. We first estimated it by the Monte Carlo minimum of $∥\tilde{A}\delta ∥/∥\delta ∥$ over pairs $\left(\theta_{1},\theta_{2}\right)$ drawn from the declared support prior with lognormal amplitudes. Extending the sampling depth from 500 to 256,000 pairs per city (30 cities) lowers the 30-city mean of this minimum monotonically, from 0.510 through 0.452 (4000), 0.410 (16,000), and 0.375 (64,000) to 0.347 (256,000), roughly linearly in logN and without any sign of flattening (Supplementary Table S1). We then ran a projected-gradient search on the unit sphere within the sign pattern of the best sampled pairs (coordinates only in $\mathrm{supp}\theta_{1}$ nonnegative, only in $\mathrm{supp}\theta_{2}$ nonpositive, shared coordinates free), which keeps every iterate inside D(C). It finds feasible directions with gain 0.076 on average (median 0.080, range 0.023–0.169), only 1.1–1.3× the sampled signed gain (median ratio 1.13), and the value decreases further with more starting points. The worst-case cone gain on these operators is therefore at most of the same order as the signed gain. The directions that attain it combine nearby collinear atoms with one sign in $\theta_{1}$ and the other in $\theta_{2}$, and they have negligible probability under the prior, which is why random sampling misses them. Consequently, no practical number of Monte Carlo samples makes the sampled *minimum* stable, and the order-of-magnitude increase reported in the submitted version was an artifact of insufficient sampling. We withdraw that claim. The worst-case bound (9) remains loose on near-collinear road dictionaries even when the nonnegativity constraint is used.

*A convergent, prior-typical diagnostic.* The quantity that random sampling does estimate consistently is a *quantile* of the gain distribution under the declared prior. The 1st-percentile cone gain is 0.608 on average and is stable in sampling depth: relative to 64,000 pairs, its city-level deviation averages 2.2% at 500 pairs, 0.8% (maximum 2.6%) at 4000, and 0.4% (maximum 1.2%) at 16,000 pairs. About 4000 pairs per operator therefore suffice for approximately stable estimates (within 3% for every city), and 16,000 for 1%. We chose the 1st percentile as the smallest quantile that is estimated stably with a few thousand samples; the 5th percentile (0.705) converges faster but describes less extreme configurations. This prior-typical gain is a descriptive diagnostic, not a guarantee of any kind. Proposition 1 is a worst-case statement, and substituting a quantile into (9) does not yield a bound that holds with probability 0.99: the quantile is taken over pairs of prior-drawn source configurations, not over the estimation error for a given truth, and deriving a probabilistic version of the bound is an open problem. We use the quantile only to compare operators, i.e., to describe how well each separates the configurations that the declared prior considers plausible. The value 0.076 found by local search is itself only an upper bound on the worst-case cone gain: it decreases with the number of restarts (Supplementary Section S3: 200 restarts per city; in a six-city check, 2000 restarts lowered it by a further 0.002–0.06). Three quantities therefore appear in this paper and are kept distinct: the worst-case gain, the only one for which (9) is a guarantee, whose true value is unknown: sampling and local search give only upper estimates (about 0.06–0.08), and because no certified lower bound is available, our numerical computations do not certify the error bound of (9); the prior-typical 1st-percentile cone gain (0.61, convergent), used descriptively to compare operators; and the sampled signed gain (0.005–0.09 in Section 7.2 and Section 7.4), retained only as an inexpensive relative diagnostic of how wind coverage or dictionary changes alter column similarity.

A further property concerns sensitivity to dictionary misspecification: $\alpha_{C,B}$ depends on the *assumed* operator alone, and dictionary misspecification enters (9) only through ρ in the numerator. Every diagnostic computed from $\tilde{A}$ is therefore provably blind to off-dictionary sources, which is why Section 7.2 needs a truth-free residual and reports an increased residual despite an approximately unchanged gain.

### 4.4. Generic Column Separation Under Wind Diversity

For an infinite-domain Gaussian impulse model the response of a compactly supported atom $\mu_{j}$ at receptor *z* under episode wind $v_{r}$ is $A_{rj}=\left(g_{\kappa \tau }*\mu_{j}\right)\left(z-v_{r}\tau \right)$. Supplementary Theorem S1 shows that if the $\mu_{j}$ are linearly independent, the P×P matrix formed by *P* episodes is invertible for Lebesgue-almost every wind tuple. The result establishes uniqueness, not conditioning, and requires R≥P episodes, whereas our cities have P=50–80 atoms and at most R=24 episodes; it is therefore not the justification for the empirical recoveries—sparsity and nonnegativity are—but it identifies wind diversity as the mechanism that separates source columns. The R≪P extension, a restricted-isometry property over the nonnegative cone, is open; we estimate a convergent prior-typical quantile of the cone gain instead (Section 4.3).

## 5. MIST Inversion and Source-Oriented Placement

### 5.1. Convex Mixed-Source Inversion

The implemented estimator profiles a smooth episode-specific background and solves

(10) $$\begin{array}{cc} \hat{\theta }=\underset{\theta \geq 0}{\arg\;\min}\; & \frac{1}{2}{∥P_{B}^{⊥}\Sigma^{-1/2}\left(y-A\theta \right)∥}_{2}^{2}\\ \; & +\lambda_{p}{∥\theta_{\mathrm{pt}}∥}_{1}+\lambda_{l}{∥\theta_{\ln}∥}_{1}\\ \; & +\frac{\lambda_{G}}{2}\theta_{\ln}^{⊤}L_{\mathrm{road}}\theta_{\ln}+\frac{\lambda_{2}}{2}{∥\theta ∥}_{2}^{2}. \end{array}$$

All penalties are set relative to data-dependent scales and held fixed across every experiment, city, and mismatch level rather than tuned per case: $\lambda_{p}=\lambda_{l}=0.008\;\lambda_{\max}$ with $\lambda_{\max}={∥{\tilde{A}}^{⊤}P_{B}^{⊥}\tilde{y}∥}_{\infty }$ (the smallest penalty that zeroes the unregularized solution), $\lambda_{2}=10^{-5}{∥\tilde{A}∥}_{2}^{2}$, and $\lambda_{G}=10^{-6}{∥\tilde{A}∥}_{2}^{2}$. Columns are normalized before penalization. At this value the road Laplacian term is numerically inactive for the sparse truths studied here; it is retained because the estimator is also intended for the spatially extended congestion regime, and pilot runs on disjoint seeds showed that aggressive corridor smoothing spreads a localized source along its corridor. Nonnegative $\ell_{1}$ terms are linear, so (10) is a convex bound-constrained quadratic program, solved with projected L-BFGS-B. The dictionary supplies the central structural restriction: Section 7.1 shows that the unregularized nonnegative least-squares special case, which Proposition 1 covers, performs as well as or better than (10) under matched transport.

### 5.2. Source-Information Placement with a Semantic Support Prior

The placement criterion we test is Bayesian D-optimality specialized to sparse mixed supports and future weather. For support *T* and weather scenario ω, let $Q_{i,T}^{\left(\omega \right)}⪰0$ be the source Fisher-information increment of receptor *i* after any *bundle-local* nuisance has been eliminated, $\Lambda_{T}≻0$ a prior precision, and 

(11) $$F\left(D\right)=E_{T∼\pi ,\;\omega ∼\Pi }\;\left[\log\det\left(\Lambda_{T}+\sum_{i\in D}Q_{i,T}^{\left(\omega \right)}\right)-\log\det\Lambda_{T}\right],$$

approximated with a fixed Monte Carlo set of 96 sampled supports and maximized greedily. Two variants are compared: an *unstructured* version with π uniform over atoms (all-source D-optimality), and a *semantic-prior* version in which π concentrates industrial candidates near plausible zones and road segments near corridors (MIST-ID). Under additive information the objective is monotone submodular and greedy selection attains the (1−1/e) guarantee (Supplementary Theorem S2); with a *shared* background nuisance the exact source information is a Schur complement whose log-determinant need not be submodular (Supplementary Section S4), so the guarantee is a design idealization. The explicit support prior allows its effect to be evaluated: Section 7.3 and Section 7.5 find that it does not improve on the unstructured version and is harmful when misspecified, providing no support for this refinement in the tested settings.

## 6. Experimental Protocol

### 6.1. Research Questions

The experiments are organized as a component-effect study. We quantify the contribution of each modeling decision, proceeding from source representation to receptor layout:

- **RQ1 (source space):** How much does the mixed point–line source model matter, separating *sector coverage* (are road emissions representable at all?) from *atom geometry* (line measures versus densified point atoms), and does its advantage survive within-family and cross-kernel transport mismatch?
- **RQ2 (dictionary fidelity):** What happens when the true sources are *not* representable in the candidate dictionary, and does any truth-free diagnostic warn the analyst?
- **RQ3 (instrument quality):** Does emission inversion survive low-cost sensor noise, persistent calibration drift, and detection limits?
- **RQ4 (excitation):** Does wind-direction coverage improve a computable identifiability quantity and inversion accuracy?
- **RQ5 (placement):** Do source-oriented and field-oriented receptor designs produce measurably different outcomes; does the distinction survive comparison against the strongest field-oriented rule; and how much does it cost when the design’s support prior is misspecified?
- **RQ6 (controlled release):** In controlled real releases, do weather-conditioned source-information subsets improve known-origin rate estimation, and does any advantage persist for a *fixed* network chosen without seeing the episode meteorology?

**Statistical conventions (used throughout).** All randomized results use 30 independently generated cities, and the city is the unit of inference. Three reporting conventions are used and each table caption names the one it uses: (i) *table cells* give means with 95% normal confidence intervals over cities; (ii) *paired differences* between methods, and the spans in the component-ranking table of Section 7, give 30-city paired percentile bootstrap intervals (20,000 resamples); (iii) *significance* uses the exact two-sided Wilcoxon signed-rank test on paired city-level errors. Paired comparisons share city, source, evaluation weather, and noise realization. With 30 pairs the smallest attainable exact two-sided *p* is $1.9\times 10^{-9}$ (all cities favor one method); any *p* below $10^{-8}$ is reported as $p<10^{-8}$. For RQ4 a Spearman correlation is computed within each city and the 30 correlations are tested across cities. An exploratory, not pre-specified, RQ5 contrast averages budgets 5, 7, and 9 within each city. Holm correction is applied within each family of three metrics (source NRMSE, support AUPRC, field NRMSE) per baseline contrast. The tests added in revision (mixing height and inflow, segment length, matched severity, placement at 4% noise) are not corrected for multiplicity and should be read as exploratory; their conclusions rest on effect sizes and bootstrap intervals rather than on individual *p*-values.

**On comparing effect sizes across decisions.** The component ranking compares source-error spans, so it is only as meaningful as the ranges over which each factor is varied. We fix those ranges from external evidence—sensor grades from published noise and drift magnitudes [29,30,31,32], wind coverage from a nearly unidirectional sector to a full rose, off-dictionary displacement of 1.5 km (comparable to candidate spacing) and an unmodeled area source carrying 35% of emitted mass, and placement over the seven criteria of Section 6.3—and report beside each range-dependent span the narrowed span of a deployment with tighter control of the corresponding factor (see the component-ranking table in Section 7).

### 6.2. Synthetic Cities and Forward Model

The domain is 20×20 km. Candidate point sources are sampled inside the boundary. Roads form horizontal and vertical corridors split into normalized line atoms. The placement study contains 18 point candidates, 32 road atoms, 40 candidate receptors, four active points, and seven active road segments. Semantic support probabilities concentrate point emissions near two industrial zones and road emissions near central corridors; truth and MIST-ID supports are independent draws from this declared prior.

The exact-model generator evaluates the finite-window two-dimensional advection–diffusion Green function

(12) $$K_{v}\left(q,s\right)=\int_{\tau_{\min}}^{\tau_{\max}}\frac{e^{-\lambda \tau }}{4\pi \kappa \tau }\exp\;\left(-\frac{{∥q-s-v\tau ∥}^{2}}{4\kappa \tau }\right)d\tau ,$$

using 24 age quadrature points over 0.08–3 h. Line responses use nine deterministic quadrature points. We set κ=1 km^2^/h and λ=0.08 h^−1^. The decay constant (*e*-folding time 12.5 h, the order of daytime ${\mathrm{NO}}_{x}$ or SO_2_ removal) acts within the 0.08–3 h window only as a mild down-weighting of the oldest contributions; it is not a reactive-chemistry model.

*Why this operator is used and what it omits.* Equation (12) is a vertically integrated (well-mixed layer) two-dimensional advection–diffusion kernel with uniform wind, a single constant diffusivity, and first-order loss. It is chosen deliberately as the *inverse* operator for three reasons: it has a closed-form, differentiable dependence on wind, so that column similarity and the restricted gain can be computed exactly for thousands of operators; it is the operator for which Proposition 1 and Supplementary Theorem S1 can be stated; and, because every component of the benchmark shares it, differences between design decisions are not confounded by differences in transport fidelity. Its idealizations are substantial and are, in decreasing expected importance for urban ${\mathrm{NO}}_{x}$: (i) no vertical structure, so that mixing-height variability, plume rise, and the near-ground versus elevated release distinction are absent; (ii) no stability dependence of dispersion; (iii) no buildings, street canyons, or urban-canopy drag; (iv) spatially uniform wind within an episode; (v) no lateral boundary inflow beyond the affine background; and (vi) no chemistry beyond linear loss. Rather than assume these are second order, the benchmark generates observations with models that contain them while the inverse keeps (12): stability classes B–F and ground-reflected vertical structure (RLINE-lineage generator), street-canyon confinement, episode-varying mixing height, non-affine regional inflow, wind speed regime, and a diurnal emission cycle (Section 7.7). The design-factor ranking is therefore evaluated *under* transport misspecification, not under the idealized operator alone.

Wind speed can affect the comparison through plume residence time and directional response, so we evaluate it explicitly. The primary protocol draws 0.8–1.9 m/s, representing low-wind conditions: low speeds lengthen residence time, sharpen directional dependence, and are the conditions of exceedance episodes. Because a diffusion-dominated regime could favor a diffusive inverse, Section 7.7 repeats the coverage and geometry comparisons at moderate (1.9–3.9 m/s) and normal (3.1–5.0 m/s) speeds, with the age window scaled inversely with speed so that transport length stays comparable (the conservative convention), and reports larger coverage ratios and geometry advantages.

Likewise κ is a single isotropic diffusivity with no stability-class dependence, so the primary results are a fixed-stability, open-terrain idealization; Section 7.7 adds an RLINE-lineage generator with Pasquill classes B–F and a screening-level street-canyon operator. For RQ1, RQ2, RQ3 (reference grade), and RQ5, observation-noise standard deviations are 4%, 7%, 4%, and 7% of signal RMS and background-basis coefficient standard deviations 15%, 20%, 20%, and 20%. Each research question uses a disjoint block of city seeds.

For placement, 24 independently sampled climatology winds construct each fixed design. Eight new winds generate observations and the reported GP spatial-interpolation metric. No evaluation wind enters the site-selection operator. The first mismatch generator changes diffusivity to 1.22, decay to 0.055, uses finer quadrature, and perturbs wind vector, plume rotation, and anisotropy. The stronger OSSE instead generates truth with an independently implemented steady Gaussian-plume kernel, episode-varying spread, and perturbed wind, while inversion retains the finite-age isotropic Green operator in (12).

### 6.3. Baselines, Designs, and Metrics

Inverse baselines are same-dictionary nonnegative least squares (NNLS), nonnegative Tikhonov inversion, a point-only sparse inverse that omits all road atoms, and two *densified-point* baselines that separate road geometry from source-sector coverage. A point-only dictionary cannot represent a line source at all, so its failure is partly definitional. The densified baselines instead replace each road segment by K∈{1,3} point atoms at equally spaced interior nodes—a point-atom approximation to a distributed road source—invert with the identical estimator over the enlarged point dictionary, and then sum the recovered node coefficients back to the segment for scoring. K=1 isolates the effect of collapsing a line to its midpoint; K=3 provides a denser approximation with three times as many road unknowns. The comparison against these baselines, not against the point-only inverse, is what isolates the value of the line-measure geometry itself. Placement baselines are random, spatial *k*-center, GP posterior variance with an RBF kernel, greedy GP mutual information with the same kernel [20], D-optimality over all source parameters, and D-optimality on the true support as a diagnostic. The last is not an error oracle because D-optimal volume is not identical to sparse-estimator NRMSE. We include both GP rules because posterior variance is a weaker heuristic and can yield a larger apparent mapping–inversion tradeoff than mutual information.

We report normalized source error $∥\hat{\theta }-\theta ∥/∥\theta ∥$, support area under the precision–recall curve (AUPRC), top-*k* recall, nominal-forward field NRMSE, and GP interpolation field NRMSE. Under mismatch, nominal-forward error compares $A_{\mathrm{nom}}\hat{\theta }$ with $A_{\mathrm{truth}}\theta$, so it does not grant the estimate access to the truth operator. The empirical restricted gain is the minimum singular value over 160 randomly sampled 2s-column submatrices after projecting out the episode background and normalizing columns. Because only a subset of supports is searched, it is an optimistic upper estimate of the true worst-case gain, not an exact restricted-isometry computation.

### 6.4. Prairie Grass Controlled Releases

Prairie Grass contains known near-ground SO_2_ releases observed on arcs at 50, 100, 200, 400, and 800 m [55]. We use the official Aarhus University workbook. After requiring complete release and meteorological metadata, the evaluation contains 66 releases and 7901 concentration measurements.

The 66 releases occur on 29 dates and share one campaign source location, so we split by date with five-fold GroupKFold, calibrate six spread parameters on training dates, treat the origin as known, and estimate only its nonnegative rate on held-out releases. Two protocols are run. In the *weather-conditioned* protocol, 8, 12, or 20 receptors are chosen per release (random, *k*-center, GP variance, candidate-source D-optimality on a 9×9 grid, or known-source sensitivity) after observing that release’s meteorology; this represents forecast-conditioned activation or mobile sampling. In the *fixed-network* protocol, one design per fold is chosen by greedy log-determinant maximization over information matrices averaged on training releases only and reused unchanged on every held-out release—the direct analogue of (11). Inference uses paired within-date contrasts over 29 date clusters and is conditional on the five fold calibrations. Because arcs and grid are centred on the known source, the data cannot validate blind localization. The campaign is also rural, short-range (≤800 m, roughly 25 times smaller than the 20 km urban domain), single-source, and uses SO_2_ as a passive tracer, so it tests only whether weather-conditioned receptor selection improves rate estimation when the transport model is approximately right; transfer to urban scales is not claimed. Full protocol details, sampler-dropout handling, and tables are in Supplementary Section S5.

### 6.5. Off-Dictionary Sources

The synthetic and OSSE experiments above share the correct candidate dictionary, so they do not assess emissions that lie outside the candidate dictionary. We therefore add 30 cities in four regimes. *Candidate-aligned* reproduces the matched reference. *Displaced points* moves every active point source 1.5 km off its candidate location in a random direction, so no column is correct. *Omitted active road* generates truth from the full dictionary but deletes one *active* road atom from the inversion dictionary. *Unmodeled area source* adds a diffuse cloud of twelve off-grid weak sources carrying 35% of total emitted mass, present in the truth only. Inversion always uses the nominal candidate dictionary and the nominal transport operator. Alongside recovery metrics we record two quantities computable *without* the truth: the sampled restricted gain of the inversion operator and the background-projected relative residual $∥P_{B}^{⊥}\left(y-A\hat{\theta }\right)∥/∥P_{B}^{⊥}y∥$. These diagnostics are evaluated for their ability to detect misspecification without known emissions.

### 6.6. Low-Cost Sensor Grades

Observation noise of 4–7% of signal RMS represents reference-grade analysers, not low-cost nodes. We therefore repeat the budget-9 placement task over four sensor grades, holding geometry and emissions fixed: reference (4% noise, no drift), calibrated low-cost (10%, 5%), typical low-cost (20%, 12%), and uncalibrated or aged (30%, 20%). Table 1 anchors each magnitude to its literature source and lists the error mechanisms that are not modeled. This experiment uses the same city generator, budget, wind protocol, and background level (20% of signal RMS) as the placement study of Section 7.5, and additionally includes GP mutual information as a third design. Its reference grade uses 4% noise and a disjoint seed block, so its reference-grade MIST-ID error (0.383) differs from the 7%-noise budget-9 value reported in Section 7.5 (0.451); comparisons should be made within each table (The two tables share protocol but not seeds or noise level and are not replicates of one another).

Two truth regimes separate instrument effects from design-prior effects: *matched-prior*, in which the truth support is drawn from the declared semantic prior MIST-ID optimizes for, and *misspecified-prior*, in which it is drawn uniformly over atoms while MIST-ID still assumes the semantic prior. Drift is modeled as a per-sensor multiplicative gain error, $y_{ri}^{\mathrm{obs}}=g_{i}\;{\left(A\theta \right)}_{ri}+{\left(B\eta \right)}_{ri}+\varepsilon_{ri}$, i.e., applied to the source-generated signal, with $g_{i}=1+\sigma_{d}z_{i}$, $z_{i}∼N\left(0,1\right)$, drawn once per sensor and held *constant across the episodes of a campaign*. Three considerations motivated this choice. (i) *Time scale.* Reported low-cost gain drift accumulates over weeks to months [31,32,34], whereas a campaign pools episodes of a few hours over a few weeks; over that window the dominant residual error after the last co-location is a near-constant sensitivity offset of each unit, and $\sigma_{d}$ (5–20%) is set to the between-unit gain spread reported after or without such calibration (Table 1). (ii) *Identifiability.* A constant gain persists across all episodes and is not reduced by pooling, whereas zero-mean fluctuations that vary from episode to episode are partly averaged by the pooled fit. Its network-common part is exactly confounded with overall source magnitude (a global scale ambiguity), while its sensor-specific part distorts the spatial pattern of the observations and is therefore misassigned between sources rather than absorbed by a single scale factor. We therefore expect, but do not prove, that constant gain is among the more harmful structures of equal magnitude; Section 7.7 tests this empirically. (iii) *Testability.* Section 7.7 replaces it at matched per-sensor standard deviation by a random walk, independent per-episode gains, a network-wide shared component (ρ∈{0,0.5,1}), and gains driven by episode wind direction or speed; none is significantly worse. Not represented are additive baseline (zero) drift, temperature- and humidity-dependent response, cross-sensitivity (e.g., O_3_ interference on electrochemical NO_2_ cells), and nonlinear response; additive drift that is common to the network is absorbed by the episode background offset, but sensor-specific additive drift is not, and these mechanisms are listed as untested in Table 1 and Section 8. Grades with drift also impose a detection limit that censors readings below 2–10% of signal RMS to zero (roughly 2–10 µg m^−3^ at our emission scale, the order of reported limits of detection for low-cost electrochemical NO_2_ and optical PM nodes); substituting LOD/2 for censored values changes mean source error by at most 0.004.

## 7. Results

Table 2 summarizes the component effects: the source-error span between the worst and best setting of each decision with all others held fixed. Discrete modeling choices and range-dependent factors are reported separately because their spans have different interpretations. The two *discrete* rows (does the source space include traffic; are roads line measures or densified points) have no free perturbation range, so their spans are the entire effect of a modeling choice. The *range-dependent* rows are as large as their declared worst setting, so a narrowed span for a deployment with tighter control of the corresponding factor is reported beside them, and no single rank is assigned across the two blocks.

The two groups show different dependencies. Within the discrete block, coverage (0.742) exceeds geometry (0.163–0.410). Within the range-dependent block, dictionary fidelity and sensor grade are tied under the declared ranges (Δ=−0.000, [−0.154,0.144]), followed by wind excitation, all three moving source error by 0.61–0.68 against 0.126 for the entire placement span; once narrowed, sensor grade (0.118) and wind excitation (0.094) fall to the order of placement, so the larger effects correspond to substantial instrument error and limited weather coverage. For the discrete modeling choices, the *ordering* is preserved under every stress test in Section 7.7, but the effect *magnitude* depends on transport fidelity—under the RLINE-lineage generator the coverage span compresses to 0.119–0.163, the order of the placement span, and the advantage moves to support identification (AUPRC gap 0.24–0.28). The spans are measured under the protocol of the corresponding research question, so coverage (4% noise, ten *k*-center receptors) and placement (7% noise, budget 9) are not obtained under identical noise. Repeating the placement study at 4% noise on the same 30 cities (Supplementary Section S6.6) gives a GP-variance-to-source-information difference of 0.075 ([−0.006,0.158]) instead of 0.126, and a full range among the six practical criteria (the true-support design is a diagnostic) of 0.148 ([0.061,0.234]) instead of 0.147. The noise difference therefore does not inflate the ratio of coverage to placement effects. The placement span itself depends on which criteria are admitted; restricted to mutual information and source-information design, it decreases to 0.020. The dictionary-fidelity span survives halving the misspecification severity (0.384 and 0.342 over 30 fresh cities), Narrowing the ranges of the other factors leaves the placement span itself unchanged but reduces their own spans: with calibrated sensors (0.118) and moderate wind excitation (0.094) they are of the same order as placement (0.126), so the comparison holds only for the declared ranges.

### 7.1. RQ1: Source-Sector Coverage Dominates Under Matched Transport; Atom Geometry Is a Second-Order Refinement

The source space is the most consequential decision in our work, with two distinct contributions: whether the dictionary covers the traffic sector at all, and whether roads are best represented as line measures.

*Coverage.* Under matched transport, the mixed point–road model attains 0.111 source NRMSE against 0.853 for an otherwise identical facility-only point dictionary—a factor of 7.7. Under within-family perturbation of diffusivity, decay, wind, rotation, and anisotropy the two give 0.260 and 0.861 (3.3×), and under the cross-kernel plume observing-system simulation experiment (OSSE), 0.496 and 0.917 (1.8×). The advantage narrows as transport error grows but never reverses. Part of this gap is definitional—a point-only dictionary has no column that can represent a line source—so it should be read as the cost of omitting the traffic sector, which is exactly what a facility-oriented inventory does, not as evidence for line measures specifically.

*Geometry.* The densified-point baselines isolate the contribution of road-atom geometry. Replacing each road segment by its midpoint (K=1) recovers much of the loss, reaching 0.521 under matched transport rather than 0.853, and three nodes per segment (K=3) reach 0.275. Line measures remain better at every mismatch level: 0.111 versus 0.275 and 0.521 when matched, 0.260 versus 0.478 and 0.570 under perturbation, and 0.496 versus 0.571 and 0.634 under the OSSE. The paired advantage over K=3 is −0.163, −0.218, and −0.075 source NRMSE (Wilcoxon $p<10^{-8}$, $p<10^{-8}$, and $p=2.2\times 10^{-3}$), i.e., a median ratio of 2.6×, 1.8×, and 1.2×, achieved with one third of the road unknowns. Covering the traffic sector is thus worth up to 7.7×, whereas the line-measure geometry itself is worth a further 1.15–2.6× with fewer parameters.

**Figure 2:**
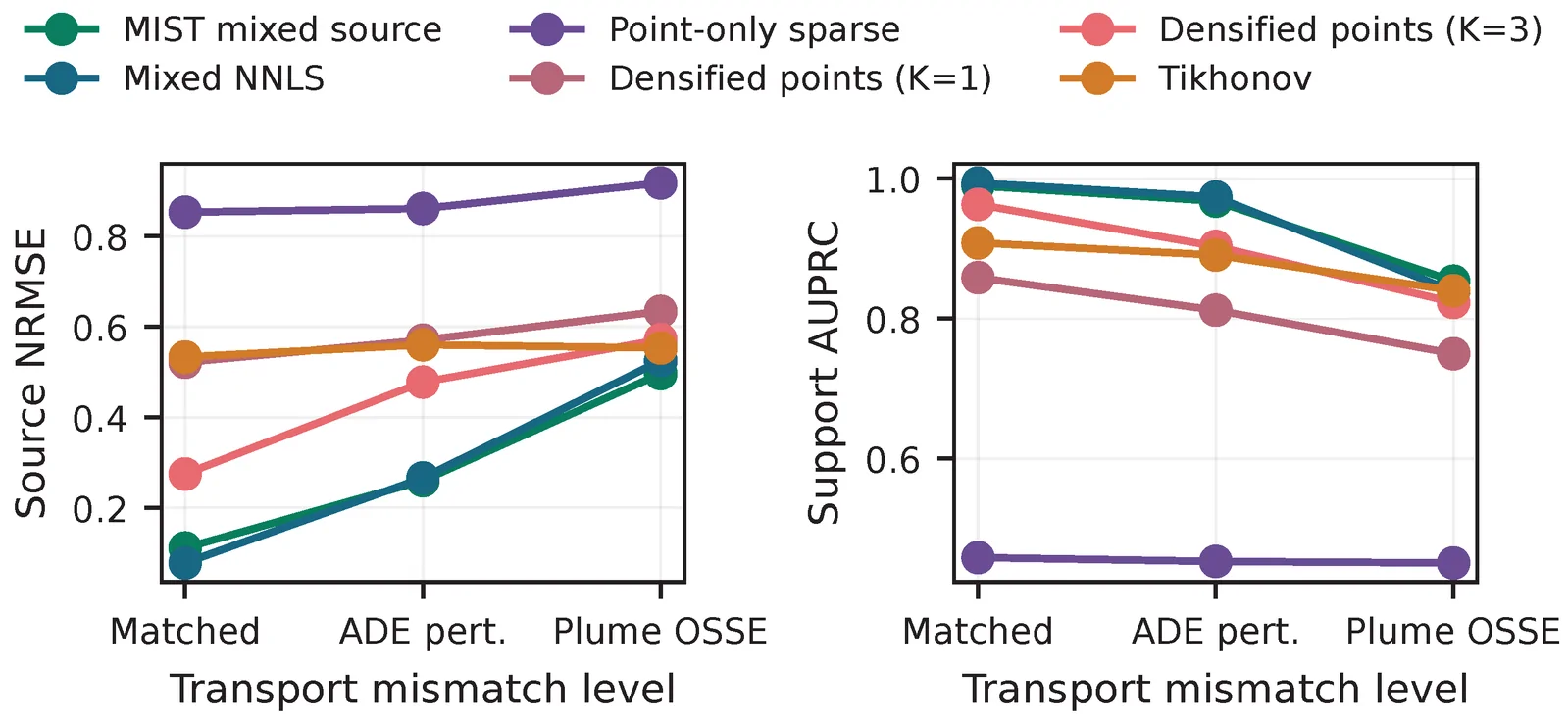
Recovery from matched transport through perturbed advection–diffusion to an independently implemented steady-plume OSSE. Line-measure estimators retain lower source error than both the facility-only point dictionary and the densified-point approximations of the same roads. Markers are means over 30 cities.

Figure 2 and Table 3 detail the OSSE across all six estimators. The estimator effect is small—MIST is lower than same-dictionary NNLS in 27 of 30 cities by a mean 0.029—and under matched transport absent (unregularized mixed NNLS, the estimator that Proposition 1 actually bounds, attains 0.078 against 0.111 for the regularized estimator behind Table 2), so the regularization is not what produces the recovery; the dictionary is, since the 0.392 gap between mixed NNLS and the facility-only model is thirteen times the estimator gap.

### 7.2. RQ2: Off-Dictionary Sources Break Recovery, and the Residual Detects It

Table 4 reports the four dictionary regimes. Against a candidate-aligned reference of 0.083 source NRMSE and 1.000 support AUPRC, displacing the active point sources 1.5 km off grid raises source error to 0.756 and lowers AUPRC to 0.722, and an unmodeled area source carrying 35% of emitted mass gives 0.766 and 0.745. Deleting one active road atom is less damaging (0.276, 0.975) because neighboring collinear segments absorb the missing emission—an aliasing that misassigns emissions along the corridor. The displaced and area-source penalties, roughly 0.68, are more than five times the 0.126 placement span of Section 7.5.

The sampled restricted gain is unchanged across regimes (0.0678–0.0730), as Section 4.3 predicts, whereas the background-projected relative residual rises from 0.061 when aligned to 0.110, 0.176, and 0.276—a factor of 1.8–4.5. Table 5 turns the residual into an alarm: across 30 further cities the aligned regime is the null, the threshold is its 95th or 90th percentile, and detection is reported at each sensor grade of Section 7.3, since noise also inflates the residual. At reference and calibrated low-cost grades the residual has high detection performance (AUC 0.95–1.00; detection 0.83–1.00 at 5% false alarms). At typical low-cost grade it still detects displaced sources (AUC 0.93) but the area source becomes marginal (AUC 0.72), and with uncalibrated nodes the area source is essentially undetectable (AUC 0.59) because the aligned residual itself has risen to 0.39; normalizing by the grade’s noise-floor residual, as co-location would allow, recovers part of the loss (AUC 0.80 at typical grade). Both residual-based detection and emission inversion deteriorate as calibration quality declines. With 30 null cities per grade the TPR columns are coarse; AUC is the more stable summary.

### 7.3. RQ3: Low-Cost Sensor Noise and Persistent Gain Drift Outweigh Design Choice

Table 6 repeats the budget-9 placement task across sensor grades under the protocol of Section 7.5. Source error grows from 0.383 (reference analyser) to 0.502, 0.770, and 1.067 as noise and persistent drift increase, while support AUPRC falls from 0.896 to 0.581. At typical low-cost quality the source error is comparable to the emission vector itself, and the reference-to-uncalibrated span of 0.683 is four times the placement span. Persistent multiplicative drift is the mechanism: constant across episodes, it is not averaged away by weather diversity and acts as a structured ΔA term amplified by $1/\alpha_{C,B}$ in (9). Source-oriented siting is therefore worth optimizing only after calibration is controlled.

The noise model isolates gain error and is not a complete low-cost sensor error budget. Electrochemical NO_2_ and optical PM nodes are additionally subject to temperature and humidity cross-sensitivity, ozone interference, nonlinear response, and spatially correlated common-mode responses [29,30,31]. Because a gain that co-varies with episode meteorology would be correlated with the transport operator rather than merely persistent, it could in principle be more damaging than the constant gain used here. Section 7.7 tests this directly: at matched magnitude, gains driven by wind direction, wind speed, or a network-wide wind-driven component are no worse than constant gain, so the instrument effect is not specific to constant gain among the tested meteorology-independent and wind-correlated structures; species interference and nonlinear response remain unsimulated.

Table 6 also provides two observations relevant to RQ5. First, the placement advantage declines as sensor quality degrades: the paired MIST-ID-minus-GP-variance source difference is −0.008[−0.084,0.068], −0.096[−0.159,−0.033], −0.051[−0.136,0.034], and −0.008[−0.124,0.109] across the four grades, so only the calibrated low-cost grade separates from zero and the results show no consistent advantage. Second, the design performance also depends on prior accuracy: in the misspecified-prior regime the ordering at reference grade reverses, MIST-ID attaining 0.466 against 0.386 for GP variance, a paired penalty of +0.080 ([0.007,0.156]) that persists in point estimate at the degraded grade (+0.088, [−0.023,0.200]). A geometric rule cannot be misspecified in this way. Because the cost of a wrong prior overlaps the interval of the entire placement span (0.126, [0.050,0.206]), placement is a coarse rather than a fine decision: the wrong criterion class and the wrong prior cost roughly the same modest amount, and both are smaller than the source-space and instrument effects. Table 6 reports the matched-prior regime.

### 7.4. RQ4: Wind Coverage Predicts Recoverability

Supplementary Figure S1 shows a monotonic increase in the sampled, nuisance-profiled gain as angular coverage expands. From $15^{\circ }$ to $360^{\circ }$, mean mutual coherence falls from 0.998 to 0.962, sampled gain rises from 0.0054 to 0.0912, and source NRMSE falls from 0.682 to 0.076. Computing a separate six-condition Spearman correlation in each city gives median ρ=−0.943; a two-sided Wilcoxon test across the 30 city correlations gives $p=1.27\times 10^{-6}$. This city-level analysis avoids treating the six repeated conditions as independent. The largest practical improvement occurs before full circular coverage: $140^{\circ }$ already reduces mean NRMSE to 0.170.

Two limitations apply to this result. The reported gain is a sampled *signed* gain, so its relative trend is more informative than its absolute value, and as a worst-case guarantee (9) is vacuous (Section 4.3): even at the upper estimates α≈0.06–0.09 (the true worst-case gain can only be smaller) the bound would demand projected noise below 1% of emitted mass for a source error near 0.2, whereas the 4% noise applied here yields 0.076; the convergent prior-quantile gain (0.61) is ten times larger, but it carries no guarantee and is used only descriptively. Even at $360^{\circ }$ coherence remains high because adjacent road measures are close, which is what the uniqueness statement of Supplementary Theorem S1 omits.

### 7.5. RQ5: Placement Matters as a Criterion Class, Not as a Refinement

**Figure 3:**
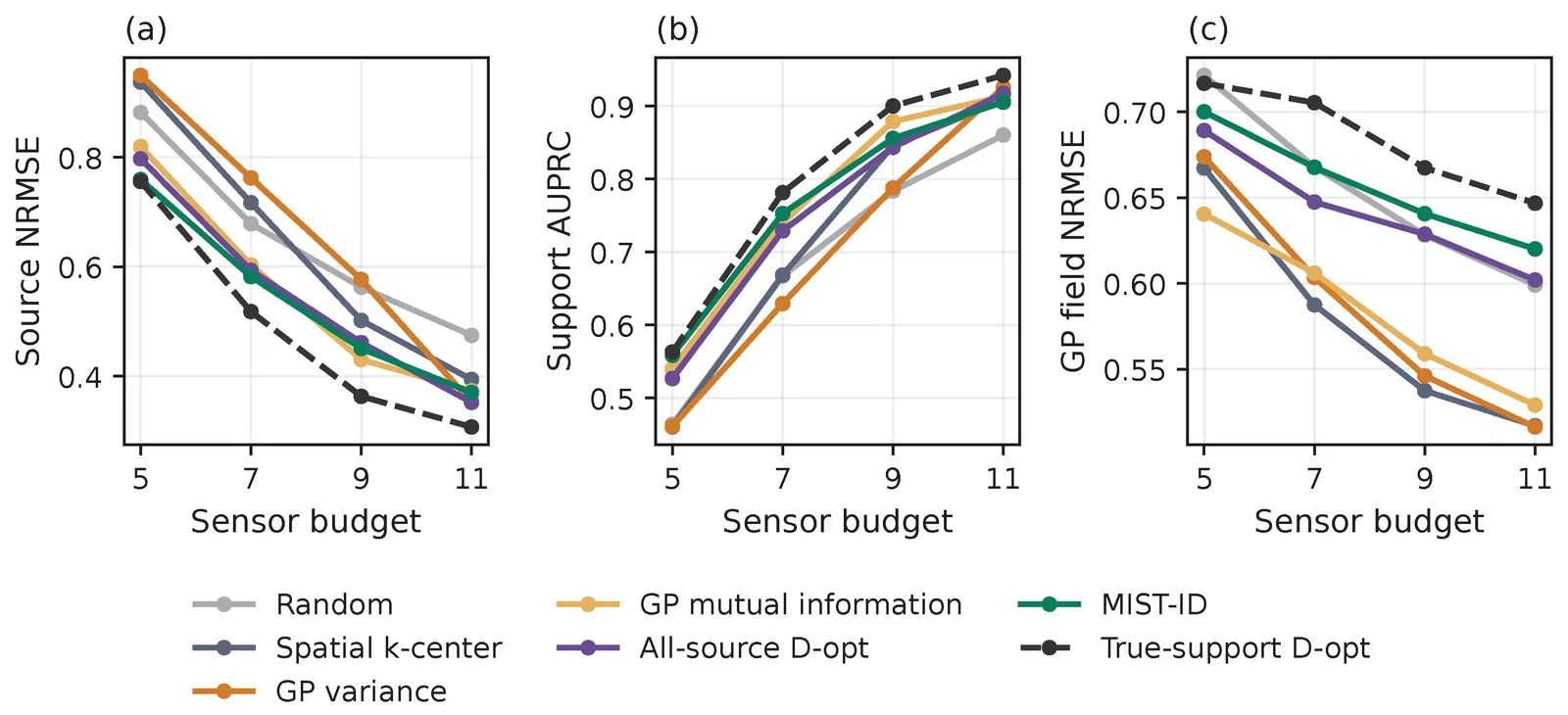
Receptor-budget tradeoff over 30 cities as a function of sensor budget: (**a**) source NRMSE (lower is better); (**b**) support AUPRC (higher is better); (**c**) GP field NRMSE (lower is better). Markers are means over cities. MIST-ID is optimized for source information and outperforms GP posterior variance on source metrics, whereas GP mutual information is competitive with MIST-ID for source recovery while remaining better for field reconstruction. The mapping–inversion tradeoff is therefore specific to the variance heuristic, not to field-oriented design in general.

Figure 3 and Table 7 compare designs under held-out weather. At budget 9, MIST-ID obtains 0.451 source NRMSE and 0.856 support AUPRC, versus 0.577 and 0.788 for GP posterior variance. The concentration result reverses: the fixed GP interpolator attains 0.546 interpolation-field NRMSE versus MIST-ID’s 0.641. In the exploratory within-city aggregate over sparse budgets 5, 7, and 9, the MIST-ID-minus-GP-variance paired differences are −0.165 for source NRMSE (95% bootstrap interval [−0.218,−0.113]; Holm $p=9.72\times 10^{-6}$), +0.096 for support AUPRC ([0.048,0.144]; $p=1.60\times 10^{-3}$), and +0.062 for interpolation-field NRMSE ([0.027,0.097]; $p=2.77\times 10^{-3}$). The improvement in source recovery is accompanied by an increase in field-interpolation error.

The choice of field-oriented baseline is decisive. Greedy GP mutual information [20] attains 0.430 source NRMSE and 0.879 support AUPRC at budget 9—slightly *better* than MIST-ID on both source metrics—while also achieving lower field error (0.559); over sparse budgets the MIST-ID-minus-mutual-information source difference is −0.020 ([−0.063,0.022], Holm p=0.76) and only the field-error penalty remains significant (+0.068, $p=2.19\times 10^{-3}$). Posterior variance concentrates receptors near domain boundaries, where plumes are weak and nearly parallel, whereas mutual information retains interior coverage that is informative about sources as well. These results do not establish a general advantage for source-oriented design over field-oriented design. They show that variance-based siting, common in practice, is specifically harmful for emission inversion and not interchangeable with mutual-information siting. The advantage is also confined to budgets below the active-support size of 11: at budget 11 MIST-ID and GP variance give 0.370 and 0.353 (p=0.73).

Three further comparisons delimit the finding. MIST-ID does not significantly outperform all-source D-optimality over budgets 5–9 (−0.020, [−0.054,0.017], p=0.30). Probing the semantic prior in three further settings chosen to favor it (per-budget contrasts, the cross-kernel OSSE, and an 80-atom dictionary with sparser truth) on an independent 15-city seed set (Supplementary Table S8), *no probe among twelve survives Holm correction* (smallest $p_{\mathrm{Holm}}=0.15$), and the prior is nominally *worse* on source magnitude at budgets 9 and 11 (+0.105–+0.133). When the truth support is drawn uniformly instead of from the declared prior (Section 7.3), MIST-ID is worse than GP variance by 0.080: a source-information design trades robustness for prior information a deployment cannot usually guarantee. The RQ5 results support the following placement choices: avoid variance-based siting, treat mutual-information and unstructured source-information designs as interchangeable, and prioritize source-space coverage and instrument quality in further design work. The true-support diagnostic illustrates the tradeoff between mapping and emission inversion: knowing the true support gives the best source NRMSE (0.363) yet the *worst* GP-interpolation error (0.667), because receptors that resolve a few emitting atoms cluster in emitting sub-regions.

### 7.6. RQ6: Known-Origin Controlled-Release Stress Test

**Figure 4:**
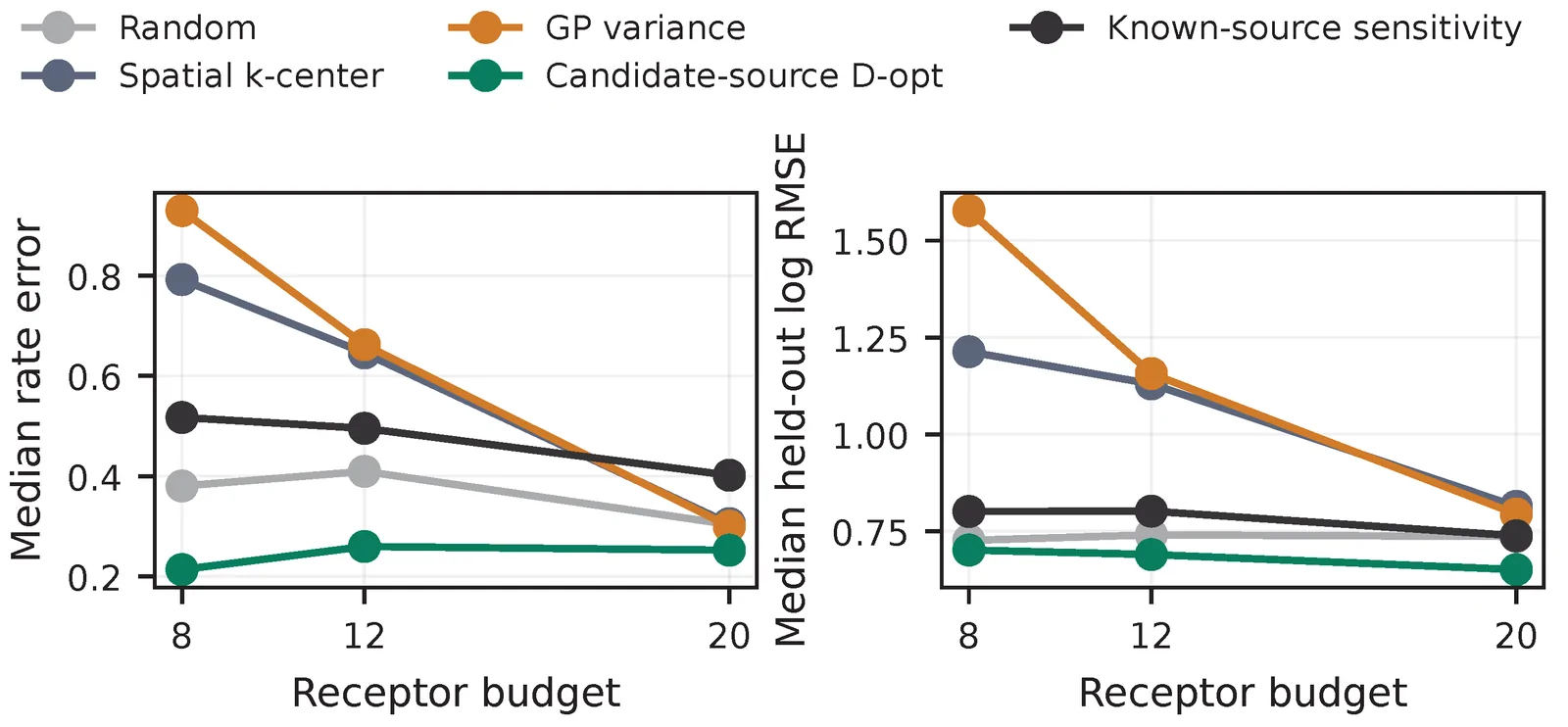
Date-grouped five-fold validation on 66 Prairie Grass controlled SO_2_ releases from 29 dates. Receptors are selected after observing episode meteorology, and rate is estimated at the known campaign origin. Curves report release medians; date-cluster tests condition on fitted fold calibrations.

Figure 4 reports the weather-conditioned protocol. With eight receptors, candidate-source D-optimal selection has 0.213 median relative rate error against 0.930 for GP variance (within-date contrast −0.595, nested fold-and-date bootstrap [−0.728,−0.404]); at budget 20 the two converge to 0.252 and 0.300. Per-release results and the remaining baselines are in Supplementary Tables S3 and S9.

Under the fixed-network protocol the effect disappears: at budget 8 the fixed source-information design attains 0.663 median rate error against 0.497 for fixed GP variance, with a paired contrast of +0.187 ([−0.112,0.387], p=0.080), and +0.133 (p=0.33) and +0.018 (p=0.38) at budgets 12 and 20. A fixed sampler set cannot follow the wind, and the training-averaged objective concentrates samplers in the climatologically most-sampled sector. The apparent benefit therefore comes from conditioning on episode meteorology, not from the criterion. For the fixed urban network that motivates this paper, RQ6 contributes a null result: it supports forecast-conditioned activation or mobile sampling and provides no evidence that a fixed installed network gains from a source-information criterion.

### 7.7. Stress Tests of the Two Discrete Orderings

Table 8 summarizes five stress tests, and Table 9 adds a sixth (mixing height and regional inflow), each designed so that it could have reversed the coverage or geometry ordering; the full protocols, tables, and discussion are in Supplementary Section S6. Three findings are common to all of them. Neither ordering reverses under these transport and emission perturbations, with the segment-length exception noted in Section 7.8 for the geometry ordering; their *magnitude* is a property of matched transport; and the compression is confined to emission magnitude, leaving support identification largely intact.

*Wind speed.* The primary protocol draws low speeds (0.8–1.9 m s^−1^), which could favor a diffusion-dominated inverse. Repeating RQ1 at moderate (1.9–3.9) and normal (3.1–5.0 m s^−1^) speeds, with the age window either scaled with speed or held fixed (Supplementary Table S2), increases the coverage ratio and geometry advantage: the coverage ratio rises from 7.7× to 9.5–9.6× and the geometry advantage from 0.163 to 0.198–0.222, because faster advection produces less overlapping footprints. The low-wind setting is therefore conservative.

*RLINE-lineage generator.* A line-source model in the RLINE/AERMOD lineage—numerical line integration, ground-reflected Gaussian vertical structure, and Pasquill-class-dependent $\sigma_{y},\sigma_{z}$—implemented independently of the inverse, with mean column norms matched to remove the unidentifiable transport scale, produces the largest transport-mismatch errors among the tested generators. The coverage span decreases from 0.742 to 0.163 (class D) and 0.119 (classes B–F varying per episode), i.e., 1.23× and 1.15×, and the geometry advantage shrinks to +0.040 (p=0.09) and +0.056 ($p=3.2\times 10^{-3}$). The support-AUPRC gap, however, remains 0.24–0.28: severe transport misspecification degrades emission-magnitude estimation much more than source-support identification. Unmodeled stability variation increases inversion error.

*Street-canyon confinement.* A screening-level operator amplifies road-atom responses within a canyon half-width and attenuates them beyond it. Mixed-source error rises to 0.403 and 0.535 under moderate and strong confinement and the coverage span falls to 0.335 and 0.175, with both orderings retained at $p<10^{-8}$ and support AUPRC essentially untouched (0.97–0.99 versus 0.46–0.51).

*Drift structure.* Replacing the constant per-sensor gain of Section 7.3 with a random walk, independent per-episode drift, or a network-wide shared component (ρ∈{0,0.5,1}) at matched magnitude changes source error by at most 0.03 and never significantly; none of these meteorology-independent structures was significantly more harmful than constant gain. Table 10 adds three *meteorology-correlated* gains at the same per-sensor standard deviation: $g_{ri}=1+\sigma \;a_{i}z_{r}$ with $a_{i}∼N\left(0,1\right)$ and $z_{r}$ the standardized cosine of the episode wind direction or the standardized wind speed, and a network-wide $g_{ri}=1+\sigma z_{r}$. None was significantly more harmful than constant gain: at calibrated grade the MIST-ID differences from constant gain are +0.006, +0.018, and +0.008 (p≥0.56), and at typical low-cost grade they are −0.075, −0.052, and −0.124 (p=0.11, 0.13, 0.002). A wind-correlated gain acts on each receptor as a rescaling that varies smoothly with the episode, which the episode-wise background profiling partly absorbs, whereas a constant gain is confounded with source magnitude in every episode. Spatially patterned common-mode responses that are not wind-driven remain untested.

*Mixing height and regional inflow.* The two-dimensional operator has no vertical structure and only an affine background, so we generated observations with two effects it cannot represent (Table 9). In a well-mixed layer, surface concentration scales as $1/h_{r}$; we multiplied each episode’s source signal by $h_{\mathrm{ref}}/h_{r}$, with $h_{r}$ lognormal (coefficient of variation 0.5) or proportional to wind speed, so that low-wind episodes are also shallow. Regional inflow was added as a non-affine boundary term $a_{r}\exp\left(-d_{\mathrm{up}}\left(x\right)/L\right)$, decaying from the upwind domain edge with L=6 km and amplitude 30% or 60% of the signal RMS. Unmodeled mixing-height variability is among the largest error sources in our work: mixed-source error rises from 0.099 to 0.41–0.53, more than three times the placement span, and support AUPRC falls from 0.99 to 0.84–0.90. A per-episode scale factor is confounded with source magnitude in exactly the way the gain drift of Section 7.3 is. Regional inflow is less harmful (0.13–0.19), because most of it projects onto the background basis. In every scenario, coverage (0.38–0.73) and geometry (+0.15 to +0.17) remain significant at $p<10^{-5}$, and the background-projected residual rises from 0.06 to 0.26–0.36 under mixing-height variability, within or above the range produced by the off-dictionary scenarios of Section 7.2 (0.11–0.28), so the truth-free alarm would flag these cases. The damage is largely remediable. Dividing each episode’s observations by an external mixing-height estimate with 15% lognormal error (typical of ceilometer- or reanalysis-derived boundary-layer depth) lowers mixed-source error from 0.528 to 0.248 and from 0.414 to 0.240 and restores support AUPRC to 0.98; with 30% error the recovery is partial (0.425). This correction is idealized in two respects: it assumes that surface concentration scales exactly as $1/h_{r}$, whereas a changing boundary layer also alters vertical dispersion and plume shape (partly a footprint error), and it assumes unbiased, episode-wise independent errors, whereas reanalysis boundary-layer depth can be systematically biased in urban areas. The recovery reported here is therefore an upper bound on what external boundary-layer data can deliver. The residual also tracks the severity of transport error: within each city, across the nine scenarios, its Spearman correlation with source error averages 0.82 (city-bootstrap 95% interval [0.77,0.86]; minimum 0.53; positive in all 30 cities, Wilcoxon $p=1.7\times 10^{-6}$). Computing the correlation within cities avoids treating the 270 city–scenario pairs as independent. The alarm therefore detects transport misspecification as well as dictionary misspecification. Mixing height should be supplied from reanalysis or ceilometer data, or estimated as an episode scale parameter in the nuisance vector η, before emission rates are interpreted.

*Diurnal traffic cycle.* Assigning each pooled episode a random hour and scaling true road intensities by a two-peak profile, while the inverse keeps q=1, increases mixed-source error from 0.087 to 0.290 and 0.349 at road CV 0.41 and 0.52, mostly through road and neighbouring point atoms; support AUPRC stays above 0.93 and the facility-only baseline stays at 0.86–0.87. At realistic amplitude the shared-intensity simplification alone costs 0.2–0.26, larger than the placement span, so campaigns pooling day and night should stratify episodes or use q>1 with a periodic basis, which (4) admits.

### 7.8. Real Road and Industrial Geometry from OpenStreetMap

The synthetic generator places roads on orthogonal corridors and industrial candidates around two zones, and this regularity may affect the observed orderings. To evaluate this dependence, we built a candidate dictionary from real geometry: a 20×20 km domain centred on Chengdu (30.62^∘^ N, 104.07^∘^ E), with motorway, trunk, and primary roads from OpenStreetMap chopped into chords of target length 2 km (mean realized length 1.64 km, because many OSM ways are shorter than the target) and thinned to 60 line atoms, and landuse=industrial polygons merged within 1.5 km into the 25 largest area-weighted clusters as point candidates (Supplementary Figure S2). The semantic prior is proportional to road class and to industrial area. Emissions, transport, receptors, and noise remain synthetic and follow the RQ1 and RQ5 protocols exactly, with 30 independent truth, wind, and noise draws on this *single* geometry; the intervals in Table 11 therefore reflect truth, wind, and noise variability but not geometry variability, and the experiment is a real-geometry OSSE, not a real-data validation. Two idealizations remain even in the geometry: secondary and residential roads are excluded, so a real deployment would face exactly the omitted-source condition of Section 7.2 unless the dictionary were extended; and the industrial clusters are land-use polygons, not permitted stacks.

Table 11 reports the outcome. The coverage effect remains substantial on real geometry: mixed-source error is 0.210 against 0.939 for facility-only points (span 0.729, $p<10^{-8}$), with support AUPRC 0.985 against 0.361. The geometry comparison differs from the synthetic layout: on real chords the midpoint densification (K=1) is *better* than K=3 (0.270 versus 0.348), because 2 km chords of an irregular network are short enough that a single midpoint atom already captures the footprint, and additional nodes only add collinear unknowns. Line measures remain best (advantage 0.060 over K=1, $p=2.6\times 10^{-7}$; 0.138 over K=3), but the geometry benefit is bounded by segment length and shrinks toward zero as segments become short relative to the plume scale. The placement ranking reproduces the synthetic one: MIST-ID, all-source D-optimality, and GP mutual information are indistinguishable (0.472, 0.490, 0.497; p=0.67 and 0.75), whereas GP variance is again specifically harmful (0.660, $p=1.2\times 10^{-4}$) and *k*-center intermediate (0.592). Neither the two-zone industrial layout nor the orthogonal corridors is therefore what drives the reported orderings.

*Sensitivity to segment length.* The 2 km chord length is a discretization choice, and the line-measure advantage could depend on it. Supplementary Table S6 varies it in two ways. On the Chengdu geometry, the dictionary was rebuilt at target chord lengths of 1.0, 1.5, 2.0, and 3.0 km (60 line atoms each, RQ1 protocol, same 30 draws); the 2.0 km row reproduces Table 11. Because OSM ways are split at junctions, realized mean lengths span only 1.06–1.85 km, so we also re-split the synthetic corridors into atoms of 1.55–18.6 km. In that experiment the *physical* truth is fixed on the finest 1.55 km sub-chords, with three active traffic stretches of 3–9 km and heterogeneous intensity along the road, so that no coarse dictionary represents it exactly; source error is scored at each dictionary’s resolution (true mass aggregated into each atom), and a resolution-independent forward NRMSE is computed at all 36 candidate receptors under eight unseen winds. Three results emerge. (i) Coverage is insensitive to segment length: the facility-only dictionary fails at every length (0.93–0.98 against 0.21–0.63). (ii) The advantage of line atoms over midpoint atoms is significant at every length but grows with segment length (from +0.03 at 1–1.5 km to +0.16–+0.22 at 6–19 km), because a midpoint misplaces more mass as the segment grows. (iii) Segment length trades representation error against collinearity. When active traffic occupies only part of a long segment, a uniform line atom spreads the mass over the whole segment, and at 6–19 km three nodes per segment (K=3) then give lower source error than one line atom, although the line dictionary still gives the lower forward error. A control with homogeneous intensity within each active stretch gives nearly identical results (e.g., 0.382 versus 0.390 for line atoms and 0.224 versus 0.245 for K=3 at 6.2 km), so the effect arises from partial occupancy of long segments, not from along-road intensity variation or from the heterogeneous truth design. When the active stretch coincides with whole segments, as in RQ1, line atoms win clearly (0.111 versus 0.275). Short atoms (1.5 km) multiply nearly collinear columns, which penalizes K=3 most strongly. Error for line atoms is smallest at 1.5–3 km on both geometries. We therefore recommend chords of roughly 1.5–3 km, i.e., comparable to the plume length scale $\sqrt{\kappa \tau }$ plus advection distance over the age window, with long corridors split further where traffic is expected to occupy only part of them. The footnote to Table 2 and the deployment guidance in Section 8 are revised accordingly.

### 7.9. A Real-Data Negative Control on a Sparse Chengdu Station Subset

We also applied the pipeline to real observations on the same Chengdu domain as a *negative control*: a configuration that the ranking predicts to be insufficient for emission inversion, so that the test is whether the available diagnostics indicate that source estimates should be withheld, rather than whether inversion succeeds. The analysis uses a sparse configuration: the seven national (CNEMC) stations inside a 26×26 km box. We used their hourly NO_2_ for January–March 2024 together with hourly 10 m wind at the domain centre from the ERA5-driven Open-Meteo archive, and an OSM dictionary of 30 industrial clusters and 80 road chords built as in Section 7.8. Hours with rain or wind below 0.5 m/s were dropped; the remainder were grouped into 6 h blocks whose vector-mean wind retained at least 60% of the mean speed, giving 311 directionally steady episodes across all eight sectors (mean speed 1.3 m/s, dominated by north-easterly flow). Each episode’s observation is the per-station median NO_2_ and its transport operator is (12) with the episode’s mean wind. There is no emission truth, so evaluation is by leave-one-station-out (LOSO) prediction of the held-out station’s episode medians, with the $\ell_{1}$ strength chosen by nested LOSO on the training stations; the comparators are the facility-only and midpoint-densified dictionaries, a GP interpolator of the six training stations, and the six-station episode mean.

Table 12 shows that the physics-based inversions predict the held-out station *worse* than the GP interpolator (LOSO NRMSE 0.30 against 0.25) and no better than the episode mean (0.26); the three dictionaries are indistinguishable from one another; and the background-projected residual is 0.87 for the mixed dictionary against 0.06 in the aligned synthetic case and 0.11–0.28 under the synthetic misspecifications of Section 7.2. Split-half (odd/even day) support stability is a Jaccard index of 0.15, and a weekday/weekend refit is degenerate. Collapsing the dictionary to six industrial zones and 34 named corridors (44 unknowns) does not improve these metrics (residual 0.90, LOSO 0.31).

Because real data carry instrument noise, chemistry, and background structure absent from the simulation, the synthetic alarm thresholds of Table 5 cannot be transferred, and no real-data threshold validated against known emissions is available. Table 13 therefore reports two surrogate nulls, which test whether the observed wind and station geometry carry detectable information, with the same stations, episodes, dictionary, and estimator: *shuffled wind*, in which the 311 episode wind vectors are permuted across episodes (breaking the wind–source coupling while keeping every marginal), and *shuffled station*, in which station coordinates are permuted (breaking the geometry). The observed residual, 0.869, lies below all 100 surrogates of either kind (shuffled-wind 5th–95th percentiles 0.899–0.916; shuffled-station 0.900–0.961; permutation p<0.01). The wind-conditioned operator therefore captures a detectable but limited signal on this network. The operator explains $1-0.869^{2}\approx 24\%$ of the background-projected variance, against $1-0.908^{2}\approx 18\%$ for a wind-blind operator, and this increment does not translate into out-of-sample predictive skill (Table 12). The surrogate tests therefore indicate detectable information in the observed wind and spatial configuration, but they do not establish the accuracy of the inferred emission rates or calibrate a real-data threshold for reliable inversion. Our decision to withhold source estimates is a conservative judgement that combines three pieces of evidence, not a pre-specified threshold rule: no out-of-sample skill over simple baselines, unstable support (Jaccard 0.15), and a residual that leaves about three quarters of the background-projected variance unexplained (for reference, roughly three times the largest synthetic misspecification residual, which is not itself a transferable threshold). The tests cannot attribute this outcome to a particular cause; insufficient receptor coverage, transport misspecification, chemistry, background structure, and calibration all remain possible.

Two qualifications apply. First, the Wilcoxon tests in Table 12 are over seven station-level errors, so the smallest attainable two-sided *p* is 0.016 and the p=0.08 entries have very low power; these tests provide no evidence that the inversions predict better than the GP and do not establish equivalence. Second, the experiment cannot separate insufficient receptor coverage from substantial transport-model misspecification; the discriminating test—repeating the LOSO and surrogate protocol on a denser provincial or micro-station network in the same domain and asking whether the residual falls toward the synthetic range—requires data beyond those available for our work.

The outcome is consistent with the synthetic study. Seven receptors for 110 (or 44) candidate atoms is far below any budget at which our designs recovered sources, and the stations are sited for population-exposure monitoring, the field-oriented objective Section 7.5 identifies as least informative for emission inversion; the open-terrain, single-diffusivity operator is, for a basin city, the transport misspecification that Section 7.7 showed degrades magnitude first; and the diagnostics flagged the limitation without ground truth. The surrogate nulls show how the presence of signal can be tested on a real network, but not how to set a threshold for reliable inversion. A positive real-data test would require a denser, source-oriented, calibrated network with independently known emissions—precisely the ingredients the ranking places above placement—and remains necessary for evaluating real-data inversion accuracy.

## 8. Discussion and Limitations

### 8.1. What the Theory Establishes

Proposition 1 connects a restricted gain to error amplification, but its worst-case form is loose on near-collinear road dictionaries even on the nonnegative cone; a convergent prior-quantile gain is a descriptive diagnostic only (Section 4.3); Supplementary Theorem S1 proves structural uniqueness in a regime (R≥P) none of our experiments occupies; Supplementary Theorem S2 guarantees greedy design only once nuisance is additive or locally removable. These are complementary statements, not a single guarantee for a chemically reactive city.

### 8.2. The Mapping–Inversion Tension Is Structural

The true-support diagnostic in Table 7 illustrates the difference between the two objectives: receptors chosen with knowledge of the true support give the best source error (0.363) and the *worst* field-interpolation error (0.667). This result follows from the receptor geometry: resolving a few emitting atoms requires receptors where their plume footprints diverge, which clusters them in emitting sub-regions; interpolating a field requires receptors spread over the domain. The two objectives therefore pull toward different geometries, and any single fixed network is a compromise. Mutual-information siting is the practical resolution because it retains interior coverage that happens to be informative about sources, whereas posterior-variance siting pushes receptors to boundaries where plumes are weak and parallel, failing both objectives at once. The design implication is that an agency wanting both maps and source estimates should not expect one criterion to deliver both, but should site a mutual-information core and add source-oriented receptors only where the candidate dictionary is trusted.

### 8.3. Source Identification Degrades More Slowly than Emission Rates Under All Tested Error Types

Across the stress tests, emission-rate error rose much more than support AUPRC fell (Table 14). A natural hypothesis attributes the pattern to the *type* of model error, distinguishing perturbations that rescale the responses of correctly located atoms (canyon, diurnal cycle, inflow, mixing height) from those that distort footprints or gains (stability-dependent dispersion, displaced or unmodeled sources, sensor gain). This comparison is confounded with severity, because the rescaling perturbations studied were also the weaker ones. We therefore swept five perturbation types over severity grids on the RQ1 protocol (30 cities; 26 inversions per city). The types were two rescaling errors (mixing height, lognormal CV 0.15–1.0; episode-varying road intensity, CV 0.25–1.6), two footprint errors (episode wind-direction error, 1–30^∘^; displaced point sources, 0.25–2.5 km), and a per-sensor gain error (SD 0.05–0.45). We then compared AUPRC loss at *matched* increases in source error (Supplementary Table S7 and Section S6.6).

At matched severity, no clear difference between the tested error types was resolved. At source-error increases of 0.2, 0.3, 0.4, and 0.5, the five types lose 0.03–0.04, 0.05–0.08, 0.12–0.13, and 0.17–0.21 AUPRC, and the footprint/gain-minus-rescaling difference is at most 0.015, with every city-bootstrap 95% interval containing zero (upper limits ≤0.034). The type-based hypothesis is therefore not supported. The sweep’s footprint perturbations (wind-direction error, source displacement) are generated within the Green-function family of the inverse, so the similarity is shown for that family; the independent RLINE-lineage generator enters only through the stress-test rows of Table 14, which are consistent with it. The data indicate that, across the five tested types, identification loss followed similar patterns at matched increases in emission-rate error. Because no equivalence margin was specified in advance, this is a finding of small and unresolved differences, not a demonstration of statistical equivalence. Because reference support AUPRC is close to its ceiling (0.99), we checked whether this coupling reflects metric saturation by repeating the sweep with top-*k* recall and with a denser truth (16 instead of 8 active atoms; Supplementary Section S6.6). The similarity persists in both checks: at matched rate error, the top-*k* loss of the three classes differs by at most 0.03 in every bin. The location of any threshold, however, depends on the metric and the support size. With 8 active atoms, AUPRC loss stays below 0.05 in 90% of inversions while the source-error increase is below 0.3, but top-*k* loss does so in only 76%, and with 16 active atoms AUPRC and top-*k* loss do so in 67% and 30%. Identification therefore degrades *more slowly* than emission rates, and more slowly the sparser the truth, rather than being protected up to a fixed threshold. Street-canyon confinement is the one exception among the stress tests, with an increase of 0.42 in source error and an AUPRC loss of only 0.02: the canyon amplifies the responses of road atoms at receptors near the road, which sharpens rather than blurs their signature.

Two practical consequences follow. First, source identification and emission-rate estimation should be reported separately, because for sparse emitter sets the identified emitters remain more reliable than the rates. Second, the background-projected residual indicates which regime a deployment is in, without knowledge of the error type: over the full matched-severity sweep, its within-city Spearman correlation with the increase in source error is 0.79 (positive in all 30 cities), and as a classifier of source-error increases above 0.3 it attains AUC 0.89, 0.93, and 0.95 for rescaling, footprint, and gain errors (0.90 pooled; 0.89–0.97 with 16 active atoms). These AUCs measure discriminative ability against a truth-defined event and do not supply an operational threshold. No real-data threshold validated against known emissions is available; same-network surrogates (Section 7.9) can test whether an operator carries signal, but they do not set an accuracy threshold. Which remedy applies does depend on the error type: rescaling errors can be removed with external scale information (Table 9), whereas footprint errors require a better transport model or dictionary.

### 8.4. Is the Dominance of Source-Space Coverage an Expected Result?

Qualitatively, yes: that omitting a major emitting sector degrades an inversion is expected, and we do not present the existence of the effect as a discovery. The benchmark adds three things that are not implied by that expectation. First, it places the effect on the same error scale as decisions that receive far more methodological attention: under matched transport the coverage span (0.74) is about five times the full range among placement criteria (0.13–0.15), and calibration and dictionary fidelity are of comparable size to coverage. These relative magnitudes, rather than the sign of the coverage effect, are what inform how a monitoring campaign allocates effort. Second, the magnitude is not robust in the way the sign is: under the independent RLINE-lineage generator and under mixing-height variability the coverage span shrinks to 0.12–0.16 and 0.38–0.49, respectively, approaching the placement span in the first case, while the advantage in source identification (support AUPRC) persists. Completeness of the source space therefore matters most for deciding *which* candidates emit, and it can be partly masked in emission-rate estimates by transport error. Third, the decomposition into coverage and geometry, and the segment-length analysis, show which part of the effect is definitional (a missing sector) and which part results from the line-source representation itself. The latter is smaller and depends on segment length and on how much of each segment is active. The conclusions remain conditional on the framework: they describe how a dictionary-constrained linear inversion with a simplified transport operator behaves under the declared perturbations, not how every operational inversion system would behave.

### 8.5. Limitations

*Off-dictionary sources.* Displaced points and an unmodeled area source raise source error from 0.083 to about 0.76 (Section 7.2), and missing road emissions alias onto collinear neighbors. The supported mitigation is diagnostic—the residual alarm—not corrective; a dictionary-refinement estimator is future work.

*Diversity of urban emission sources.* The candidate dictionary contains only industrial point sources and traffic line sources. Real urban emissions also include area sources (residential and commercial combustion, cooking, construction), fugitive emissions, distributed commercial activity, and inventories that change over time. Within the present framework these enter in two ways. Sources that are spatially compact but absent from the dictionary are off-dictionary errors, and the unmodeled area source of Section 7.2 (twelve diffuse sources carrying 35% of the mass) shows that they raise source error to about 0.7 while the residual flags them. Persistent area emissions could instead be added as *area atoms* (unit-mass footprints over land-use polygons) in (3) without changing the estimator, but their columns are smoother and more collinear with the background basis and with one another, so their identifiability would be lower than that of point and line atoms. We have not evaluated such a three-type dictionary, and the reported ranking should not be extrapolated to cities where area and fugitive sources dominate the species of interest. Inventories that evolve over time correspond to q>1 or to campaign-specific dictionaries.

*Passive primary tracer.* PM_2.5_ includes secondary chemistry and regional formation, and large emission reductions can yield counterintuitive responses [56]; NO_2_/${\mathrm{NO}}_{x}$, CO, black carbon, or controlled tracers are more defensible first targets.

*Forward-operator idealizations.* The Green function assumes open terrain, a well-mixed layer of fixed depth, uniform wind, a single diffusivity, and linear loss, and episodes share one intensity per source. Mixing height is the most consequential of the effects tested: unmodeled episode-to-episode variability raises source error to 0.41–0.53 (Table 9), so the ranking was tested under, but not validated for, realistic urban boundary layers. Urban-canopy drag, building wakes resolved at street scale, spatially varying wind, and nonlinear chemistry (e.g., NO–NO_2_–O_3_ partitioning) were not simulated. Whether the ranking persists under a full urban dispersion or chemical-transport model (e.g., an obstacle-resolving or WRF–CMAQ-type generator) is an open question that this benchmark cannot answer. Section 7.7 bounds rather than removes these effects: canyon confinement and stability variation degrade magnitude heavily while preserving support identification, so quantitative rates in built-up canyons require an obstacle-resolving forward model; the diurnal cycle costs 0.2–0.26 at realistic amplitude, and the remedy (q>1 with a periodic basis, admitted by (4)) is not evaluated. An across-episode network-wide gain is an unidentifiable overall scale; wind-correlated gains are no worse than constant gain (Table 10), but common-mode responses that are not wind-driven, species interference, and nonlinear response are untested.

*Simulation-based evidence.* We emphasize that the manuscript provides no evidence that the framework performs successful emission inversion under real urban conditions. All quantitative results on urban mixed-source inversion are simulation-based; the only real-data inversion with known emissions is Prairie Grass, a rural controlled release that does not validate urban mixed-source inversion. The Chengdu geometry experiment (Section 7.8) uses synthetic emissions, and the real-data study (Section 7.9) is a negative control. Prairie Grass is rural, short-range, single-source, and known-origin; its fixed-network null is not a sampler-dropout artifact (GP variance has the fewest present samplers on 35 of 43 retained releases, so dropout biases *toward* our criterion, and varying the presence threshold from two to five changes nothing), but RQ6 validates neither blind discovery, nor a fixed urban network, nor the mixed dictionary. A positive real-data demonstration would require a dense, source-oriented, calibrated network with independently known emissions (e.g., a controlled tracer release in an urban area, or a facility with continuous emission monitoring) and remains the principal open test of this work.

*Design and instruments.* Shared transport nuisance breaks the submodular guarantee, and robust design should integrate wind and dispersion uncertainty. The semantic support prior is harmful under misspecification and we recommend unstructured D-optimality. At 20–30% noise with persistent drift the source error reaches 0.77–1.07 and placement ceases to matter; cross-sensitivity and species interference are not simulated, and the results do not endorse emission inversion from uncalibrated networks.

*Relevance to air quality management.* The framework estimates emission rates; it does not apportion concentrations to sources, which is the task that most air quality plans rely on. Its intended use is therefore upstream of source attribution: verifying or updating the facility- and road-level emission rates that forward attribution and planning models take as input, and identifying candidate sources whose inventory rates are inconsistent with observations. Whether it is useful in practice depends on conditions that our results make explicit—a complete candidate list, calibrated sensors, adequate wind diversity, and real-data diagnostics showing out-of-sample skill, stable support, and a small residual—and on validation against independently known emissions, which our work does not provide.

*Scope of the terminology.* Following Section 3.1, “source identification” and “emission-rate estimation” mean inference within a declared candidate dictionary and transport model, not causal identification of regulatory responsibility and not receptor-model apportionment. Inventories such as MIX/MEIC [57] and interventions such as COVID mobility restrictions [58,59] can provide external consistency checks, but neither is ground truth.

### 8.6. Deployment Implications

Conditional on the declared ranges, and without an analysis of costs or implementation difficulty, the measured effect sizes (Table 2) suggest the following:

1. **Cover every emitting sector.** Omitting traffic cost 1.8–7.7×; line measures add 1.15–2.6× with fewer unknowns; in our tests chords of about 1.5–3 km minimized line-atom error on both geometries, with long corridors split further where traffic occupies only part of them (Supplementary Table S6).
2. **Control calibration.** Where calibration errors are substantial, sensor grade moved source error by 0.683 against 0.126 for placement; with calibrated nodes the effect shrinks to 0.118, the order of placement, and co-location is required to constrain an across-episode network-wide gain.
3. **Supply boundary-layer scaling.** Unmodeled mixing height raised source error to 0.41–0.53; a 15%-accurate external estimate (ceilometer or reanalysis) recovers most of the loss (Table 9).
4. **Audit dictionary completeness, and report the background-projected residual with every estimate.** Off-dictionary sources cost about 0.68; the residual detects them with AUC ≥0.93 at calibrated grades without ground truth, and a rising residual should suspend source estimates.
5. **Select an appropriate placement criterion.** Mutual-information siting served both mapping and emission inversion; prefer an unstructured or geometric criterion unless the support distribution is well established. Design matters only at budgets at or below the active-support size.
6. **Report identification and rates separately, with conditional identifiability.** Identification degraded more slowly than rates under every tested error type (Section 8.3), and the residual indicates how severe the model error is; estimates should carry support scores, posterior correlations, or the prior-quantile gain, not only a heat map.

## 9. Conclusions

Our work quantified how much sensor placement affects emission-rate inversion for urban facilities and road segments from a sparse monitoring network, relative to other design decisions. To answer it, we placed five design decisions—source space, dictionary fidelity, sensor quality, wind coverage, and receptor placement—on one error scale (O1) and tested their ranking under six independent transport and emission perturbations (O2). Within the tested framework and declared ranges, placement was not the decision with the largest effect. Under matched and moderately misspecified transport, covering the traffic sector, a complete dictionary, and calibrated sensors each matter several times more than the choice among placement criteria; within placement only the criterion class matters, and the semantic support prior evaluated here provides no benefit. Line atoms outperform densified points with fewer unknowns, most clearly for chords of about 1.5–3 km. The coverage ordering (dictionaries with versus without road atoms) never reversed under the tested perturbations. The line-atom advantage persisted in the transport stress tests, whereas densified points achieved lower source error for partially active long segments (≳6 km) in the segment-length experiment, although line atoms retained the lower forward error. The magnitude of both orderings depends on transport fidelity. Swept to matched severity, five tested error types degraded source identification in similar ways and more slowly than emission rates, so the two should be reported separately (O3). Unmodeled mixing height is among the most common errors and can be corrected with external boundary-layer data. Worst-case identifiability bounds are uninformative on near-collinear road dictionaries, whereas a prior-quantile gain gives a convergent descriptive diagnostic for comparing operators. A truth-free residual detects dictionary errors and tracks transport-error severity. On a sparse Chengdu station subset, poor out-of-sample skill, unstable support, and a large residual led us to withhold source estimates; same-network surrogates showed detectable signal but cannot certify accuracy (O4). All urban inversion results are simulation-based. Where major inventory omissions, calibration errors, or unmodeled boundary-layer scaling are present, correcting them may reduce error more than switching among the placement criteria tested; we also recommend reporting source identification, emission rates, and residual diagnostics separately. Future work should test the rate–identification coupling with obstacle-resolving and chemical-transport generators, add area atoms and time-resolved intensities, and validate the pipeline on a real network with independently known emissions.

## Figures and Tables

**Table 1:** Sensor grades and the published values each magnitude is anchored to. Noise is expressed relative to episode signal RMS; drift is a multiplicative gain error.

Grade	Noise (% Signal RMS)	Gain Drift (%)	Literature Anchor and Conversion
Reference analyser	4	0	Regulatory-equivalent precision (≲2 ppb at 50 ppb NO_2_); no drift within campaign
Calibrated low-cost	10	5	Post-calibration RMSE 3–5 ppb at 30–50 ppb and few-%-per-month drift [30,31]
Typical low-cost	20	12	Uncorrected commercial nodes, $R^{2}\approx 0.6$–0.8 (≈20% relative RMSE) [29]
Uncalibrated/aged	30	20	No periodic co-location; cumulative gain drift over months [31,32]
*Not modeled:* temperature/humidity cross-sensitivity, O_3_ interference on electrochemical NO_2_ cells, and other spatially structured biases; these are correlated across co-located nodes and are not represented by independent multiplicative drift.			

Relative values are obtained by dividing the cited absolute RMSE by a typical urban episode level of 30–50 ppb NO_2_; detection limit 2–10% of signal RMS for grades with drift.

**Table 2:** Component effects: source-NRMSE span controlled by each design decision, under the ranges declared in Section 6. Each span is the mean paired difference between the worst and best setting of one decision with all others held fixed, on the same 30 cities; brackets are 30-city paired percentile bootstrap 95% intervals. Block (a) contains discrete modeling choices whose span is the whole effect; block (b) contains range-dependent factors whose *declared* span is a property of the chosen range as much as of the factor, so a *narrowed* span for a deployment with tighter control of the corresponding factor is given beside it. The last column gives the span under the most adverse stress test of Section 7.7 and Section 7.8. Footnotes: ^¶^ depends on segment length: the advantage over midpoint atoms grows with length, while partial occupancy of long segments favors *K* = 3 on segments ≳6 km (Supplementary Table S6); ^†^ area-source mass halved to 17.5%; ^‡^ calibrated low-cost (10%, 5%) → reference; ^§^ $140^{\circ }\to 360^{\circ }$; ^‖^ GP mutual information → source-information design. Under the RLINE generator the coverage span is of the same order as the placement span, although its support-AUPRC gap (0.24–0.28) is not.

Design Decision (Worst → Best Setting)	Declared Span	Narrowed Span	Most Adverse Test
**(a) Discrete modeling choices**—no perturbation range; the span is the whole effect			
Source-sector coverage: facility-only → mixed point–road (RQ1)	0.742 [0.679,0.802]	—	0.119–0.163 (RLINE)
Road atom geometry: densified K=3 → line measures (RQ1) ^¶^	0.163 [0.127,0.205]	—	0.040–0.056 (RLINE)
versus K=1 midpoint densification	0.410 [0.361,0.458]	—	0.060 (OSM chords)
**(b) Range-dependent decisions**—declared and narrowed ranges			
Dictionary fidelity: unmodeled area source → aligned (RQ2)	0.683 [0.620,0.749]	0.342 [0.302,0.383] ^†^	—
Sensor grade: uncalibrated → reference (RQ3)	0.683 [0.553,0.826]	0.118 [0.059,0.184] ^‡^	—
Wind excitation: $15^{\circ }$ → $360^{\circ }$ coverage (RQ4)	0.606 [0.552,0.659]	0.094 [0.040,0.160] ^§^	—
Placement criterion: GP variance → source-information (RQ5)	0.126 [0.050,0.206]	0.020 [−0.022,0.063] ^‖^	0.188 (OSM geometry)
Design support prior: misspecified → matched (RQ5)	0.080 [0.007,0.156]	—	—

**Table 3:** Cross-kernel plume OSSE over 30 cities (mean ± 95% normal CI). ↓, lower is better; ↑, higher is better. “MIST mixed source” is the regularized estimator (10); “Mixed NNLS” is its unregularized nonnegative least-squares special case on the same dictionary, which Proposition 1 covers; “Tikhonov” is nonnegative $\ell_{2}$-regularized inversion on the same dictionary.

Estimator	Source NRMSE ↓	Support AUPRC ↑	Field NRMSE ↓
MIST mixed source	0.496 ± 0.065	0.854 ± 0.043	0.231 ± 0.018
Mixed NNLS	0.525 ± 0.069	0.839 ± 0.050	0.225 ± 0.018
Tikhonov	0.553 ± 0.029	0.840 ± 0.033	0.275 ± 0.018
Densified points (*K* = 3)	0.571 ± 0.055	0.823 ± 0.045	0.261 ± 0.020
Densified points (*K* = 1)	0.634 ± 0.062	0.750 ± 0.054	0.264 ± 0.017
Point-only sparse	0.917 ± 0.044	0.451 ± 0.025	0.367 ± 0.027

**Table 4:** Off-dictionary robustness over 30 cities (mean ± 95% normal CI) for the regularized mixed estimator. ↓, lower is better; ↑, higher is better. The last two columns are computable without ground truth: the sampled signed restricted gain of the inversion operator (a relative diagnostic only) and the background-projected relative residual.

Regime	Source NRMSE ↓	Support AUPRC ↑	Restricted Gain	Rel. Residual
Candidate-aligned (reference)	0.083 ± 0.012	1.000 ± 0.000	0.0678	0.061
Displaced points (off-grid)	0.756 ± 0.084	0.722 ± 0.052	0.0693	0.276
Omitted active road atom	0.276 ± 0.036	0.975 ± 0.013	0.0730	0.110
Unmodeled area source	0.766 ± 0.066	0.745 ± 0.050	0.0693	0.176

**Table 5:** Detection operating characteristic of the background-projected residual (30 cities). The aligned regime is the null; TPR is the detection rate at 5% and 10% false-alarm rate; the last column normalizes the residual by the noise-floor residual implied by each grade’s noise model.

Sensor Grade	Regime	Aligned Resid.	Misspec. Resid.	AUC	TPR@5%FA	TPR@10%FA	AUC (Noise-Norm.)
Reference (4%, none)	Displaced points	0.061	0.286	0.99	0.97	0.97	0.97
Reference (4%, none)	Area source	0.061	0.179	1.00	1.00	1.00	1.00
Calibrated low-cost (10%, 5%)	Displaced points	0.141	0.313	0.99	0.93	0.93	0.99
Calibrated low-cost (10%, 5%)	Area source	0.141	0.221	0.95	0.83	0.90	0.99
Typical low-cost (20%, 12%)	Displaced points	0.274	0.384	0.93	0.73	0.73	0.93
Typical low-cost (20%, 12%)	Area source	0.274	0.317	0.72	0.23	0.40	0.80
Uncalibrated (30%, 20%)	Displaced points	0.393	0.475	0.84	0.40	0.50	0.88
Uncalibrated (30%, 20%)	Area source	0.393	0.418	0.59	0.20	0.23	0.63

**Table 6:** Sensor-grade sensitivity at budget 9 over 30 cities (mean ± 95% normal CI), matched-prior regime.

Sensor Grade (Noise, Drift)	Source NRMSE ↓			Support AUPRC ↑		
	MIST-ID	GP MI	GP Var.	MIST-ID	GP MI	GP Var.
Reference analyser (4%, no drift)	0.383 ± 0.058	0.350 ± 0.060	0.391 ± 0.062	0.896 ± 0.041	0.916 ± 0.034	0.899 ± 0.040
Calibrated low-cost (10%, 5%)	0.502 ± 0.043	0.524 ± 0.053	0.598 ± 0.059	0.835 ± 0.037	0.831 ± 0.037	0.781 ± 0.055
Typical low-cost (20%, 12%)	0.770 ± 0.064	0.769 ± 0.056	0.821 ± 0.069	0.692 ± 0.045	0.666 ± 0.051	0.626 ± 0.064
Uncalibrated/aged (30%, 20%)	1.067 ± 0.115	1.076 ± 0.109	1.074 ± 0.071	0.581 ± 0.049	0.561 ± 0.042	0.533 ± 0.047

**Table 7:** Synthetic placement at budget 9 (mean ± 95% normal CI); field error uses the fixed GP interpolator.

Design	Source NRMSE ↓	Support AUPRC ↑	GP-Interp. NRMSE ↓
True-support D-opt	0.363 ± 0.056	0.900 ± 0.036	0.667 ± 0.038
GP mutual information	0.430 ± 0.066	0.879 ± 0.043	0.559 ± 0.031
MIST-ID	0.451 ± 0.050	0.856 ± 0.035	0.641 ± 0.034
All-source D-opt	0.461 ± 0.057	0.843 ± 0.045	0.629 ± 0.036
Spatial k-center	0.502 ± 0.062	0.846 ± 0.049	0.537 ± 0.023
Random	0.563 ± 0.058	0.783 ± 0.047	0.628 ± 0.027
GP variance	0.577 ± 0.064	0.788 ± 0.047	0.546 ± 0.023

**Table 8:** Stress tests of the coverage and geometry orderings (30 cities per test; mean source NRMSE). Neither ordering reverses; compression is confined to magnitude. Coverage span: facility-only minus mixed-source NRMSE. Geometry span: densified K=3 minus line measures (positive favors lines). AUPRC gap: mixed minus facility-only support AUPRC. *^a^* p=0.09; all other spans $p<10^{-2}$. Full tables in Supplementary Section S6.

Stress Test	Setting	Mixed Err.	Cov. Span	Geom. Span	AUPRC Gap
None (reference)	low wind, matched transport	0.111	0.742	+0.16	0.53
Wind speed	moderate/normal wind	0.085/0.082	0.719/0.708	+0.20/+0.22	—
RLINE-lineage generator	class D/classes B–F	0.710/—	0.163/0.119	+0.04 *^a^*/+0.06	0.28/0.24
Street canyon	moderate/strong confinement	0.403/0.535	0.335/0.175	+0.08/+0.05	0.48/0.46
Drift structure	random-walk, per-episode, shared, wind-correlated	≤0.03 from constant gain; wind-correlated ≤ constant			
Diurnal traffic cycle	road CV 0.41/0.52, q=1 inverse	0.290/0.349	≈0.57/0.52	—	0.51/0.48

**Table 9:** Unmodeled mixing-height variability and regional boundary inflow (RQ1 protocol, 30 cities; mean source NRMSE). Perturbations act on the truth only; the inverse keeps (12) and the affine background. MLH: mixing height (CV of the realized factor ≈0.3 when proportional to wind speed); inflow amplitude relative to signal RMS; Resid.: background-projected relative residual. Spans are paired differences from the mixed dictionary, with Wilcoxon *p* in parentheses. The reference row uses an independent noise stream and therefore differs slightly from 0.111. Lower block: observations divided by an external mixing-height estimate with the stated lognormal error. AUPRC: support AUPRC of the mixed dictionary.

Truth Perturbation	Mixed	AUPRC	K=3	Fac.-Only	Coverage (*p*)	Geometry (*p*)	Resid.
Reference	0.099	0.991	0.282	0.853	0.754 (<$10^{-8}$)	+0.183 (<$10^{-8}$)	0.059
MLH, CV 0.5, independent	0.528	0.838	0.681	0.909	0.381 (<$10^{-8}$)	+0.153 ($2.0\times 10^{-6}$)	0.357
MLH ∝ wind speed	0.414	0.900	0.584	0.900	0.487 (<$10^{-8}$)	+0.171 ($3.2\times 10^{-6}$)	0.263
Inflow 30%	0.125	0.993	0.299	0.856	0.731 (<$10^{-8}$)	+0.174 (<$10^{-8}$)	0.077
Inflow 60%	0.192	0.983	0.343	0.861	0.669 (<$10^{-8}$)	+0.151 ($4.7\times 10^{-8}$)	0.111
MLH ∝ speed + inflow 30%	0.423	0.897	0.589	0.908	0.485 (<$10^{-8}$)	+0.165 ($5.1\times 10^{-6}$)	0.262
MLH, CV 0.5, corrected (15% error)	0.248	0.978	0.413	0.867	0.620 (<$10^{-8}$)	+0.165 ($8.0\times 10^{-8}$)	0.141
MLH, CV 0.5, corrected (30% error)	0.425	0.927	0.554	0.910	0.485 (<$10^{-8}$)	+0.129 ($6.7\times 10^{-4}$)	0.242
MLH ∝ speed, corrected (15% error)	0.240	0.977	0.407	0.867	0.627 (<$10^{-8}$)	+0.167 ($1.9\times 10^{-8}$)	0.137

**Table 10:** Meteorology-correlated versus constant sensor gain at matched per-sensor standard deviation (budget 9, 30 cities, mean ± 95% normal CI of source NRMSE, matched-prior regime). Wind-direction and wind-speed gains use an independent per-sensor sensitivity $a_{i}$; the network-wide gain is shared by all sensors.

Sensor Grade	Design	Constant Per-Sensor	Wind-Direction-Correlated	Wind-Speed-Correlated	Network-Wide, Wind-Driven
Calibrated low-cost (10%, 5%)	MIST-ID	0.493 ± 0.039	0.499 ± 0.047	0.512 ± 0.043	0.501 ± 0.043
Calibrated low-cost (10%, 5%)	GP variance	0.651 ± 0.066	0.630 ± 0.070	0.660 ± 0.053	0.645 ± 0.072
Typical low-cost (20%, 12%)	MIST-ID	0.773 ± 0.059	0.698 ± 0.047	0.721 ± 0.078	0.649 ± 0.046
Typical low-cost (20%, 12%)	GP variance	0.903 ± 0.082	0.861 ± 0.071	0.850 ± 0.058	0.847 ± 0.065

**Table 11:** Real-geometry OSSE on the Chengdu OSM dictionary (30 truth/wind/noise draws; mean ± 95% normal CI). Δ is the paired difference from the reference method in each block (mixed source; MIST-ID); *p* is two-sided Wilcoxon.

Study	Method	Source NRMSE	Support AUPRC	Δ vs. Reference	Wilcoxon *p*
Source space	Densified points (*K* = 1)	0.270 ± 0.048	0.976 ± 0.019	+0.060	$2.6\times 10^{-7}$
Source space	Densified points (*K* = 3)	0.348 ± 0.047	0.948 ± 0.024	+0.138	<$10^{-8}$
Source space	MIST mixed source	0.210 ± 0.044	0.985 ± 0.014	+0.000	—
Source space	Point-only sparse	0.939 ± 0.051	0.361 ± 0.022	+0.729	<$10^{-8}$
Placement (budget 9)	All-source D-opt	0.490 ± 0.053	0.816 ± 0.041	+0.019	0.67
Placement (budget 9)	GP mutual information	0.497 ± 0.056	0.800 ± 0.057	+0.025	0.75
Placement (budget 9)	GP variance	0.660 ± 0.063	0.678 ± 0.053	+0.188	$1.2\times 10^{-4}$
Placement (budget 9)	MIST-ID	0.472 ± 0.057	0.832 ± 0.048	+0.000	—
Placement (budget 9)	Spatial k-center	0.592 ± 0.065	0.741 ± 0.055	+0.120	0.005

**Table 12:** Chengdu seven-station subset, January–March 2024, 311 steady-wind episodes: leave-one-station-out prediction of held-out median NO_2_ (7 stations). Wilcoxon *p* is over the seven station-level errors.

Predictor	LOSO NRMSE ↓	Stations Better than GP (of 7)	Wilcoxon *p* vs. GP
Mixed point–road	0.304	1	0.08
Facility-only points	0.306	1	0.08
Densified points (*K* = 1)	0.303	1	0.08
GP interpolation	0.245	0	—
Episode mean	0.261	2	0.38

**Table 13:** Same-network surrogate null for the Chengdu residual (mixed dictionary, 311 episodes, 7 stations). Each surrogate refits the estimator with permuted episode winds or permuted station coordinates; 100 permutations each.

Configuration	Residual	5th pct	95th pct	Fraction of Surrogates ≤ Observed
Observed (real wind, real stations)	0.869	—	—	—
Shuffled-wind surrogate (n=100)	0.908	0.899	0.916	0.00
Shuffled-station surrogate (n=100)	0.937	0.900	0.961	0.00

**Table 14:** Coupling between emission-rate error and source-identification loss (mixed dictionary, 30 cities). Stress tests of Section 7.2, Section 7.3, Section 7.4, Section 7.5, Section 7.6 and Section 7.7, as differences from each experiment’s own matched reference (reference source NRMSE: 0.083 for the off-dictionary rows, 0.383 for the sensor-gain row, whose reference includes 4% noise under the placement protocol and is therefore not directly comparable with the other rows, 0.087 for the diurnal test, 0.099 for the rows from Table 9, and 0.111 for the canyon and RLINE rows). The matched-severity comparison across error types is given in Supplementary Table S7.

Stress Test (Source)	ΔNRMSE	AUPRC Loss
Regional inflow, 60% (Table 9)	+0.09	0.01
Diurnal traffic cycle, CV 0.52 (Section 7.7)	+0.26	0.07
Mixing height ∝ wind speed (Table 9)	+0.32	0.09
Street canyon, strong (Section 7.7)	+0.42	0.02
Mixing height, CV 0.5 (Table 9)	+0.43	0.15
RLINE-lineage, class D (Section 7.7)	+0.60	0.28
Displaced point sources, 1.5 km (Section 7.2)	+0.67	0.28
Unmodeled area source (Section 7.2)	+0.68	0.26
Uncalibrated sensor gain (Section 7.3)	+0.68	0.32
RLINE-lineage, classes B–F (Section 7.7)	+0.69	0.35

## Data Availability

Our work uses synthetic observations generated under the protocols in Section 6, the Prairie Grass workbook provided by Aarhus University, OpenStreetMap geometry, hourly Chengdu NO_2_ observations from the China National Environmental Monitoring Centre, and hourly wind from the Open-Meteo ERA5 archive. The original Prairie Grass workbook is obtained from its provider, which states no explicit reuse license. OpenStreetMap data are © OpenStreetMap contributors (ODbL); CNEMC station coordinates are from https://air.cnemc.cn/ (accessed on 2 September 2026). Experimental settings, processing procedures, and aggregate results are described in the main text and Supplementary Material. The simulation and analysis code and the processed data are available from the corresponding authors upon reasonable request.

## Supplementary Materials

The following supporting information can be downloaded at: https://www.mdpi.com/article/10.3390/s26216924/s1, Table S1: Monte Carlo convergence of the cone-restricted gain on the 30 reference operators; Table S2: Wind-speed regimes under two age-window conventions; Table S3: Prairie Grass fixed-network contrast; Table S4: Independent versus network-wide correlated drift at matched magnitude; Table S5: Diurnal road cycle in the truth versus a q=1 inverse; Table S6: Sensitivity to road-segment length; Table S7: AUPRC loss at matched increase in source NRMSE, by perturbation type; Table S8: Semantic support prior probes; Table S9: Prairie Grass known-origin task at budget 8; Figure S1: Wind-direction coverage, empirical identifiability, and recovery over 30 cities; Figure S2: Candidate dictionary built from OpenStreetMap for a 20×20 km domain centred on Chengdu. The Supplementary Material contains the finite-rank non-identifiability remark (Section S1), the statements of Theorems S1 and S2 and the proofs of Proposition 1 and Theorems S1 and S2 (Section S2), the coherence-based sparse-identifiability derivation (Section S3), the shared-nuisance counterexample to submodularity (Section S4), the wind-window and Prairie Grass fixed-network tables (Section S5), the full stress-test protocols and results (Section S6), additional tables (Section S7), and the wind-coverage and OpenStreetMap-geometry figures (Section S8). Reference [60] is cited in the Supplementary Materials.
