# Supplementary material for: Source-Space Coverage Outweighs Receptor Placement in Sparse Urban Emission Inversion Under Matched and Moderately Misspecified Transport: A Mixed Point–Road Simulation Benchmark
Source: Sensors (Basel). 2026 Nov 1;26(21):6924. doi: 10.3390/s26216924 (PMC13766552; doi:10.3390/s26216924)
Supplement: Supplementary file 1 [file sensors-26-06924-s001.zip › sensors-4589862-supplementary.pdf]

Supplementary Materials: Source-Space Coverage Outweighs Receptor Placement in Sparse Urban Emission Inversion Under Matched and Moderately Misspecified Transport: A Mixed Point–Road Simulation Benchmark

Qiang Liu, Qingmin Hou, Qin Meng, Lunda Yan, Hanyang Zhang and Ziguang Jia

S1. Finite Observations Do Not Identify an Arbitrary Nonnegative Source

Let $U \subset \Omega \times (0,T)$ be nonempty and open, suppose the admissible source class contains $C_c^\infty(U)$, and let the source-to-observation map $\mathcal{L} : X \to \mathbb{R}^M$ be linear. Since $\operatorname{rank} \mathcal{L} \leq M < \infty$ while X is infinite dimensional, $\ker \mathcal{L}$ is infinite dimensional. Nonnegativity does not remove the ambiguity: taking $M+1$ nonnegative bumps φ_j with pairwise disjoint supports in U, the $M \times (M+1)$ matrix with columns $\mathcal{L}(\varphi_j)$ has a nonzero null vector c, and $S_\pm = \sum_j |c_j|\varphi_j \pm \sum_j c_j \varphi_j$ are distinct, nonnegative, and observationally identical. This standard rank argument identifies the need for source constraints beyond those supplied by the observations.

S2. Theorem Statements and Proofs

Proposition 1 is stated in the main text. The two theorems below are referred to in Sections 4.4 and 5.2 of the main text; their assumptions differ from the experimental regime, so they establish complementary theoretical properties.

Theorem S1 (Generic wind-shift identifiability).

Let $\kappa > 0$, $\tau > 0$, receptor z, and $g_{\kappa\tau}(x) = (4\pi\kappa\tau)^{-d/2} e^{-\|x\|^2/(4\kappa\tau)}$. Let $\mu_1, \ldots, \mu_P$ be linearly independent compactly supported finite measures and $A_{rj} = (g_{\kappa\tau} * \mu_j)(z - v_r\tau)$. Then there exist P wind vectors for which the $P \times P$ matrix A is invertible; $\det A$ is a nonzero real-analytic function of $(v_1, \ldots, v_P)$; and if the joint law of $(v_1, \ldots, v_P)$ is absolutely continuous with respect to Lebesgue measure on $\mathbb{R}^{dP}$, then A has full rank with probability one.

Theorem S2 (Conditional submodularity).

If $\Lambda_T \succ 0$, $Q_{i,T}^{(\omega)} \succeq 0$, and information is additive across actions, then F in Eq. (11) of the main text is normalized, monotone, and submodular, and greedy maximization under $|D| \leq k$ returns D_{gr} with $F(D_{\mathrm{gr}}) \geq (1 - 1/e) \max_{|D| \leq k} F(D)$.

Proof of Proposition 1 (restricted identifiability and stability).

The kernel condition follows by setting $\delta = \theta_1 - \theta_2$. For the bound, profiling η gives a least-squares objective in $P_B^\perp \widetilde{A}$. Optimality at $\widehat{\theta}$, followed by the triangle inequality, yields $\left\| P_B^\perp \widetilde{A}(\widehat{\theta} - \theta^\star) \right\|_2 \leq 2 \left\| P_B^\perp \Sigma^{-1/2}(\varepsilon + \rho) \right\|_2$. Apply the definition of the nuisance-profiled restricted gain, Eq. (7) of the main text,

$$\alpha_{\mathcal{C},B} = \inf_{\delta \in \mathcal{D}(\mathcal{C}) \setminus \{0\}} \frac{\left\| P_B^\perp \widetilde{A} \delta \right\|_2}{\|\delta\|_2}.$$

Proof of Theorem S1 (generic wind-shift identifiability).

Set $f_j = g_{\kappa\tau} * \mu_j$. Gaussian convolution is injective on finite measures because its Fourier multiplier $e^{-\kappa\tau\|\omega\|^2}$ never vanishes. Hence the f_j are linearly independent. Their evaluation vectors $F(x) = (f_1(x), \ldots, f_P(x))$ must span $\mathbb{R}^P$; otherwise a nonzero vector orthogonal to every $F(x)$ would contradict function independence. Choose $x_1, \ldots, x_P$ with independent evaluations and set $v_r = (z - x_r)/\tau$. This proves existence. Each f_j

extends to an entire function on $\mathbb{C}^d$ because it is the convolution of a Gaussian with a compactly supported measure, so every entry $A_{rj} = f_j(z - v_r\tau)$ is jointly real analytic in $(v_1, \ldots, v_P) \in \mathbb{R}^{dP}$ and the determinant, a polynomial in the entries, is a real-analytic function on $\mathbb{R}^{dP}$ that is not identically zero by the existence step. The zero set of a nontrivial real-analytic function on a connected open set has Lebesgue measure zero [1].

Proof of Theorem S2 (monotone submodularity and greedy guarantee).

Fix (T, ω) and write $M_D = \Lambda_T + \sum_{i \in D} Q_i$. If $D \subseteq E$, then $M_D^{-1} \succeq M_E^{-1}$. The matrix determinant lemma expresses the marginal gain of i as $\log\det(I + Q_i^{1/2} M_D^{-1} Q_i^{1/2})$, which decreases as the set grows. Expectations and nonnegative sums preserve submodularity. The approximation ratio is the classical greedy bound.

S3. Additional Identifiability Details

For column-normalized A with mutual coherence μ, every subset T of at most $2s$ columns has Gram matrix with unit diagonal and off-diagonal magnitude at most μ. Gershgorin's theorem yields $\sigma^2_{\min}(A_T) \geq 1 - (|T| - 1)\mu$, the coherence condition stated in Section 4.2 of the main text; hence $(2s - 1)\mu < 1$ is a sufficient uniform sparse-identifiability condition. This condition is sufficient but not necessary.

Adding a weather episode cannot decrease information after known or episode-local nuisance is removed. With $\bar{A}_r = P^{\perp}_{B_r} \widetilde{A}_r$ and $G_R = \sum_{r=1}^{R} \bar{A}_r^\top \bar{A}_r$, we have

$$\ker G_R = \bigcap_{r=1}^{R} \ker \bar{A}_r, \qquad G_{R+1} = G_R + \bar{A}_{R+1}^\top \bar{A}_{R+1} \succeq G_R. \tag{S1}$$

The new episode strictly resolves an old ambiguity only if its operator is nonzero on a direction in $\ker G_R$.

Computation of the restricted gains.

The signed gain is the minimum singular value over randomly sampled $2s$-column submatrices of the background-projected, column-normalized operator; it falls from 0.078 with 20 sampled subsets to 0.064, 0.059, and 0.057 with 80, 320, and 1,280 subsets (30 cities), i.e., it has largely stabilized by the 160 subsets used in the main text. The cone-restricted gain is estimated from pairs (θ_1, θ_2) with supports drawn from the declared support prior and log-normal magnitudes, $\delta = \theta_1 - \theta_2$. Both sampled minima are upper estimates of the corresponding infima.

Non-convergence of the sampled cone minimum.

The sampled cone minimum is not a usable estimate of the infimum. Table S1 extends the sampling depth to 256,000 pairs per city: the 30-city mean minimum keeps falling roughly linearly in $\log N$. A projected-gradient search on the unit sphere, started from the sign patterns of the 200 best sampled pairs and kept inside $\mathcal{D}(\mathcal{C})$, reaches a mean gain of 0.076 (median 0.080, range 0.006–0.144), i.e. 1.1–1.3× the sampled signed gain, and a six-city check with 2000 starts lowers it further. An order-of-magnitude ratio between cone and signed gains, reported in the submitted version, is therefore withdrawn. The worst-case cone gain is of the same order as the signed gain, and the directions that attain it have negligible prior probability. By contrast, the 1st-percentile gain under the declared prior converges at the usual Monte Carlo rate (Table S1, deviations relative to 64,000 pairs). About 4000 pairs give city-level estimates within 3%, and 16,000 within 1.2%.

Table S1. Monte Carlo convergence of the cone-restricted gain on the 30 reference operators. The sampled minimum (nested stream) does not stabilize; the 1st-percentile gain does. Deviations are city-level relative differences from the 64,000-pair value.

Pairs N	Sampled minimum	1st-percentile gain	Mean |dev.| (%)	Max |dev.| (%)
500	0.510	0.608	2.2	8.5
1,000	0.491	0.609	1.8	4.9
2,000	0.470	0.610	1.3	3.6
4,000	0.452	0.608	0.8	2.6
8,000	0.434	0.607	0.6	2.1
16,000	0.409	0.608	0.4	1.2
32,000	0.389	—	—	—
64,000	0.375	0.608	0.0	0.0
128,000	0.366	—	—	—
256,000	0.347	—	—	—

S4. A Shared-Nuisance Counterexample

Let independent priors be $\theta, \eta \sim \mathcal{N}(0,1)$ and let $y_1 = \eta + \varepsilon_1$, $y_2 = \theta + \eta + \varepsilon_2$, with independent unit Gaussian noise. For $F(D) = I(\theta; y_D)$,

$$\begin{aligned} F(\{1\}) &= 0, \\ F(\{2\}) &= \tfrac{1}{2}\log(3/2), \\ I(\theta; y_2 \mid y_1) &= \tfrac{1}{2}\log(5/3). \end{aligned} \tag{S2}$$

Thus the marginal value of y_2 increases after observing y_1, violating diminishing returns. The exact shared-background Schur-complement objective therefore has no general submodular guarantee.

S5. Wind-Window and Prairie Grass Fixed-Network Tables

Table S2. Wind-speed regimes under two age-window conventions (mean source NRMSE over 30 cities). Spans are paired differences relative to the mixed model.

Age-window convention	Wind regime	Mixed	Facility-only	Coverage span	Geometry span (K=3)
Length-matched window	Low (0.8–1.9 m/s)	0.111	0.853	0.742	0.163
Length-matched window	Moderate (1.9–3.9 m/s)	0.085	0.804	0.719	0.198
Length-matched window	Normal (3.1–5.0 m/s)	0.082	0.790	0.707	0.221
Fixed 0.08–3 h window	Low (0.8–1.9 m/s)	0.111	0.853	0.742	0.163
Fixed 0.08–3 h window	Moderate (1.9–3.9 m/s)	0.078	0.811	0.734	0.186
Fixed 0.08–3 h window	Normal (3.1–5.0 m/s)	0.075	0.803	0.728	0.206

Table S3. Prairie Grass fixed-network contrast (medians). One design per fold is chosen from training dates only and reused on all held-out releases.

Fixed design (chosen on training dates only)	Budget	Rate error ↓	Held-out log RMSE ↓	Median present / releases
Fixed source D-opt	8	0.663	0.844	6 / 43
Fixed random	8	0.550	0.801	6 / 43
Fixed k-center	8	0.419	0.784	6 / 43
Fixed GP variance	8	0.497	0.805	6 / 43
Fixed source D-opt	12	0.513	0.792	9 / 50
Fixed random	12	0.441	0.822	9 / 50
Fixed k-center	12	0.422	0.822	9 / 50
Fixed GP variance	12	0.481	0.819	9 / 50
Fixed source D-opt	20	0.506	0.777	14 / 55
Fixed random	20	0.397	0.755	14 / 55
Fixed k-center	20	0.349	0.771	15 / 55
Fixed GP variance	20	0.429	0.785	14 / 55

S6. Full Stress-Test Protocols and Results

This section describes the protocols and results of the stress tests summarized in Section 7.7 and Table 10 of the main text.

S6.1 Sensitivity to Conventional Wind Speeds

The primary protocol draws episode speeds from a range corresponding to roughly 0.8–1.9 m s^{-1}, which is low for an urban boundary layer and could favor a diffusion-dominated inverse. We therefore repeated the coverage and geometry comparisons in three speed regimes—low (0.8–1.9 m s^{-1}), moderate (1.9–3.9), and normal (3.1–5.0)—holding transport *length* comparable by scaling the age window inversely with mean speed, so that the comparison controls for the residence-time advantage of low winds.

Higher wind speeds retain the coverage ordering and increase the coverage ratio. Mixed-source error falls from 0.111 at low wind to 0.085 at moderate and 0.082 at normal speeds, while the facility-only baseline also improves but far less (0.853, 0.804, 0.790), so the absolute coverage span changes from 0.742 to 0.719 and 0.708 while the ratio rises from 7.7× to 9.5× and 9.6×. The geometry comparison behaves the same way: the advantage over $K=3$ densification is 0.163, 0.198, and 0.222 across the three regimes. The mechanism is that faster advection produces more directional, less overlapping plume footprints, which decorrelates source columns and helps a line-measure dictionary more than a point one, because it is the extended atoms whose responses were most nearly collinear. We conclude that the low-wind setting is conservative with respect to the observed coverage ratios and geometry advantages, and we report the moderate regime alongside it wherever the distinction could matter. Because scaling the age window with speed while holding λ fixed also changes the fraction of emitted mass that survives to the receptor, we repeated the comparison with the 0.08–3 h window held fixed, so that faster winds simply carry plumes farther. The two conventions agree to within 0.02 on every entry (Supplementary Table S2): under the fixed window the coverage span is 0.742, 0.734, and 0.728 and the geometry span 0.163, 0.186, and 0.206 across the three regimes, so the conclusion does not depend on the windowing convention.

S6.2 An Independently Implemented RLINE-Lineage Generator

The plume OSSE of Section 7.1 of the main text uses a steady-plume generator from the same Gaussian family as the inverse. We therefore developed an independent line-source implementation in the RLINE/AERMOD/Briggs lineage—numerical line integration, ground-reflected Gaussian vertical structure, and Pasquill-stability-dependent σ_y, σ_z—from the published formulations, without reusing the inverse implementation. The inverse retains a single fixed diffusivity and therefore cannot represent stability-dependent plume shape at all. Because the two kernels carry different physical units and an overall transport-to-emission scale factor is unidentifiable from a single tracer and absorbed by calibration in any deployment, we match mean column norms before inversion. This removes the overall scale but leaves every shape difference intact, and it means the RLINE test evaluates *relative* magnitudes across atoms; absolute emission totals would additionally require a calibrated transport scale, which no single-tracer inversion supplies.

This generator produces the largest transport-mismatch errors in our work. Under neutral class D throughout, mixed-source error is 0.710 versus 0.873 for facility-only points: a coverage span of 0.163, or 1.23×, against 7.7× under matched transport. When Pasquill class varies per episode over B–F—adding variation the inverse cannot express—all errors rise and the span narrows further to 0.119 (1.15×). The *magnitude* of the coverage advantage is thus a property of the matched-transport regime. The compression, however, is almost entirely in magnitude, not in identification. Support AUPRC remains 0.711 versus 0.433 under fixed-D and 0.641 versus 0.399 under mixed stability—a gap of 0.24–0.28, essentially undiminished from the matched-transport gap of 0.53 relative to the reduction in the magnitude gap. Severe transport misspecification degrades emission-magnitude estimation more than source-support identification, which remains relevant to screening and

enforcement applications. The geometry advantage over K=3 densification also persists at $+0.040$ (fixed-D, $p = 0.09$) and $+0.056$ (mixed stability, $p = 3.2 \times 10^{-3}$), so line measures remain preferable but by a margin that this test cannot always resolve. Stability variation increases error because the inverse cannot represent it; meteorological diversity improves identifiability only along dimensions represented by the forward model.

S6.3 Street-Canyon Confinement

Our transport operator is an open-terrain Green function with no buildings, which is the single largest physical idealization in our work. A full obstacle-resolving simulation is out of scope, but the direction and rough size of the effect can be bounded with a screening-level canyon operator that confines road-atom emissions: within a canyon half-width the response to road atoms is amplified by a near-field gain and beyond it attenuated by a far-field factor, with point atoms left untouched, so the perturbation targets the road atoms evaluated in the coverage and geometry comparisons.

Mixed-source error rises from 0.111 with no canyon to 0.403 under moderate confinement and 0.535 under strong confinement, and the coverage span falls from 0.742 to 0.335 and 0.175. Both orderings nonetheless persist with unchanged significance in every regime (coverage $p < 10^{-8}$; geometry versus K=3 $+0.163$, $+0.082$, $+0.046$, all $p < 10^{-8}$), and the same magnitude-versus-support split appears as in the RLINE test: support AUPRC is 0.990, 0.987, and 0.972 for the mixed model against 0.459, 0.503, and 0.509 for facility-only points, so canyon confinement has a small effect on support identification but substantially degrades magnitude estimation. In a real street canyon the mixed dictionary should therefore be expected to identify the emitting segments but to misestimate their rates unless the forward operator resolves the geometry.

S6.4 Sensitivity to the Temporal and Spatial Structure of Sensor Drift

Section 7.3 of the main text modeled sensor drift as a per-node multiplicative gain held constant across episodes. This assumption has two consequences: constant gain is a structured perturbation that repeated weather cannot average away, but it also does not interact with the shared-intensity assumption, whereas real nodes drift over time. We therefore compared three drift models at matched per-sensor magnitude—constant, a cumulative random walk over episodes, and independent per-episode drift—so that only the temporal structure differs.

The instrument-quality result persists across the three drift models. At every sensor grade the three models are statistically indistinguishable in source error, with paired differences against the constant-gain model whose bootstrap intervals contain zero at all grades; the same holds for support AUPRC. This behavior is consistent with the estimator structure: the estimator pools episodes through a sum of per-episode normal equations, so per-episode gain fluctuations enter as additional zero-mean multiplicative noise and are averaged, whereas a constant bias is not. Constant gain is therefore expected to be at least as harmful as the episode-varying structures at fixed magnitude; the tests show no significant difference among the three, so we regard the choice as not optimistic rather than as a proven worst case.

Correlated network-wide drift, such as a shared temperature or humidity response, is the other realistic structure. Table S4 decomposes the drift variance at matched magnitude into an independent per-sensor constant and a per-episode excursion common to all sensors, with the shared fraction $\rho \in \{0, 0.5, 1\}$, at the calibrated and typical low-cost grades (a fresh seed block, so its $\rho = 0$ column reproduces Table 6 of the main text to within 0.03 rather than exactly). Shared drift is no more damaging than independent drift: MIST-ID error moves from 0.507 to 0.490 (calibrated) and 0.749 to 0.686 (typical) as ρ goes from 0 to 1, and GP variance is flat within its interval. The reason is that a gain common to all sensors within an episode rescales that episode's observation vector, which the shared-intensity inverse absorbs as a per-episode scale mismatch spread evenly over atoms rather than as a spatially structured distortion. Two limitations apply: a network-wide gain that is also

constant *across* episodes is indistinguishable from an overall emission scale and would bias every estimate by that factor without appearing in any diagnostic; and a spatially *patterned* common-mode response (for example, sensors in one district sharing a microclimate) is between the two cases tested and is not covered.

Table S4. Independent versus network-wide correlated drift at matched magnitude (budget 9, 30 cities, mean source NRMSE ± 95% CI). ρ is the fraction of drift variance shared across all sensors within an episode.

Sensor grade	Method	Independent ($\rho = 0$)	Half shared ($\rho = 0.5$)	Fully shared ($\rho = 1$)
Calibrated low-cost (10%, 5%)	MIST-ID	0.507 ± 0.054	0.505 ± 0.054	0.490 ± 0.058
Calibrated low-cost (10%, 5%)	GP variance	0.622 ± 0.060	0.631 ± 0.062	0.626 ± 0.068
Typical low-cost (20%, 12%)	MIST-ID	0.749 ± 0.067	0.715 ± 0.069	0.686 ± 0.073
Typical low-cost (20%, 12%)	GP variance	0.889 ± 0.065	0.904 ± 0.070	0.880 ± 0.061

Meteorology-correlated gains (wind-direction-driven, wind-speed-driven, and network-wide wind-driven, at matched per-sensor standard deviation) are reported in Table 8 of the main text; none is worse than constant gain. These results apply only within the assumed linear-response regime.

S6.5 Diurnal Traffic Cycle versus the Shared-Intensity Assumption

The estimator pools episodes under a single intensity per atom ($q = 1$), whereas road traffic follows a strong diurnal cycle. To quantify the resulting error, we assign each of the 18 pooled episodes a random hour of day and scale the true road intensities by a two-peak (08h, 18h) profile with unit mean and increasing amplitude, holding industrial points constant; the inverse keeps $q = 1$ and is scored against the episode-mean intensities. Random hours are the pessimistic case for a campaign: episodes confined to one traffic period would realize a smaller road CV at the same amplitude. The profile's coefficient of variation runs from 0 to 0.52, with night-time minima from 78% down to 24% of the mean, so the upper rows correspond to a network that is nearly idle overnight.

Table S5 shows a progressive increase in error with diurnal amplitude. Mixed-source error rises from 0.087 with no cycle to 0.130, 0.220, 0.290, and 0.349 as the road CV reaches 0.15, 0.29, 0.41, and 0.52; the road-atom error rises fastest (0.090 to 0.413) and, because the unmodeled temporal variation is absorbed partly by the background nuisance and partly by neighbouring atoms, point-atom error also increases (0.076 to 0.271) even though the points themselves are constant. Support identification changes comparatively little (AUPRC 0.998 to 0.930), and the facility-only baseline remains at 0.86–0.87 throughout, so the coverage ordering is unchanged. These results indicate that at a realistic road CV of 0.4–0.5 the shared-intensity simplification alone costs about 0.2–0.26 in source NRMSE—larger than the placement span and comparable to the narrowed instrument span—so a campaign pooling day and night episodes should either stratify episodes by traffic period or move to $q > 1$ with a periodic temporal basis, which the formulation in Eq. (4) of the main text already admits.

Table S5. Diurnal road cycle in the truth versus a $q = 1$ inverse (30 cities, mean ± 95% CI). Road CV is the coefficient of variation of the realized road intensities across the 18 pooled episodes; errors are against the episode-mean intensities.

Road-cycle amplitude	Road CV	Method	Source NRMSE	Road NRMSE	Point NRMSE	Support AUPRC
0.0	0.00	MIST mixed source	0.087 ± 0.021	0.090 ± 0.011	0.076 ± 0.032	0.998 ± 0.003
0.0	0.00	Point-only sparse	0.870 ± 0.049	1.000 ± 0.000	0.750 ± 0.103	0.445 ± 0.025
0.5	0.15	MIST mixed source	0.130 ± 0.022	0.155 ± 0.026	0.104 ± 0.020	0.997 ± 0.006
0.5	0.15	Point-only sparse	0.867 ± 0.047	1.000 ± 0.000	0.741 ± 0.096	0.448 ± 0.024
1.0	0.29	MIST mixed source	0.220 ± 0.035	0.260 ± 0.040	0.174 ± 0.039	0.987 ± 0.009
1.0	0.29	Point-only sparse	0.866 ± 0.046	1.000 ± 0.000	0.739 ± 0.095	0.451 ± 0.023
1.5	0.41	MIST mixed source	0.290 ± 0.049	0.342 ± 0.056	0.227 ± 0.049	0.963 ± 0.017
1.5	0.41	Point-only sparse	0.864 ± 0.045	1.000 ± 0.000	0.732 ± 0.094	0.452 ± 0.023
2.0	0.52	MIST mixed source	0.349 ± 0.057	0.413 ± 0.065	0.271 ± 0.060	0.930 ± 0.031
2.0	0.52	Point-only sparse	0.871 ± 0.045	1.000 ± 0.000	0.748 ± 0.093	0.448 ± 0.022

S6.6 Road-Segment Length and Matched-Severity Error Comparison

Segment length (main text Section 7.8). Synthetic rows: the six corridors of the RQ1 geometry are re-split into atoms of 1.55–18.6 km. The physical truth is fixed on 1.55 km sub-chords, with three active traffic stretches of 2–6 sub-chords. Source error is scored at each dictionary's resolution, with true mass aggregated into each atom; forward NRMSE is computed at all 36 candidate receptors under eight unseen winds and does not depend on resolution. A control run with homogeneous intensity within each stretch gives nearly identical values. OSM rows: the Chengdu dictionary is rebuilt at target chord lengths of 1.0/1.5/2.0/3.0 km (60 line atoms each), and truth is drawn on each dictionary.

Table S6. Sensitivity to road-segment length (30 draws per row; mean source NRMSE). Length: mean atom length. Fwd.: forward NRMSE. p: two-sided Wilcoxon.

Geometry	Length (km)	Line	K=1	K=3	Fac.-only	K=1−Line (p)	Fwd. line/K=3
Synthetic	18.60	0.628	0.811	0.546	0.963	+0.183 (1.2×10^{-5})	0.308 / 0.342
Synthetic	9.30	0.473	0.630	0.317	0.956	+0.157 (3.5×10^{-8})	0.207 / 0.234
Synthetic	6.20	0.390	0.611	0.245	0.951	+0.221 (2.3×10^{-6})	0.167 / 0.184
Synthetic	3.10	0.267	0.373	0.313	0.942	+0.106 (8.0×10^{-8})	0.077 / 0.121
Synthetic	1.55	0.275	0.303	0.427	0.933	+0.028 (0.035)	0.068 / 0.105
OSM	1.06	0.252	0.278	0.334	0.976	+0.025 (3.4×10^{-4})	—
OSM	1.40	0.227	0.258	0.351	0.978	+0.030 (0.001)	—
OSM	1.64	0.210	0.270	0.348	0.939	+0.060 (2.6×10^{-7})	—
OSM	1.85	0.262	0.326	0.404	0.933	+0.065 (5.0×10^{-5})	—

Matched-severity comparison (main text Section 8.3). Each of five perturbation types is swept over a severity grid on the RQ1 protocol (30 cities; the inverse is nominal): mixing height (episode factor, lognormal CV 0.15–1.0), episode-varying road emission (CV 0.25–1.6), episode wind-direction error (1–30°), displaced active point sources (0.25–2.5 km), and per-sensor constant gain (SD 0.05–0.45). Noise and background realizations are shared across perturbations within a city. AUPRC loss is interpolated linearly at fixed mean increases in source NRMSE; intervals are city-bootstrap percentiles (2000 resamples).

Table S7. AUPRC loss at matched increase in source NRMSE, by perturbation type. Last rows: pooled footprint/gain minus pooled rescaling types, with 95% intervals.

Perturbation type	AUPRC loss at source-error increase of			
	0.2	0.3	0.4	0.5
Rescaling: mixing height	0.031	0.052	0.122	0.192
Rescaling: road emission cycle	0.027	0.076	0.133	0.182
Footprint: wind-direction error	0.030	0.081	0.133	0.209
Footprint: displaced points	0.037	0.080	0.127	0.174
Gain: per-sensor gain	0.038	0.082	0.129	0.179
Footprint/gain − rescaling	+0.007	+0.015	+0.003	+0.003
95% interval	[-0.007, +0.021]	[-0.005, +0.034]	[-0.023, +0.027]	[-0.027, +0.034]

Metric-saturation check. Because reference support AUPRC is near 0.99, the sweep was repeated (i) scoring top-k recall and (ii) with a denser truth of 16 active atoms (6 points, 10 roads). The fraction of inversions with identification loss below 0.05 while the source-error increase is below 0.3 is 0.90 (AUPRC) and 0.76 (top-k) with 8 active atoms, and 0.67 and 0.30 with 16. Between-class differences in top-k loss at matched source-error increase are at most 0.03 in every bin for both support sizes. The residual flags source-error increases above 0.3 with AUC 0.89/0.93/0.95 (rescaling/footprint/gain; 8 atoms) and 0.90/0.92/0.97 (16 atoms); its within-city Spearman correlation with the source-error increase is 0.79 for both, positive in all 30 cities.

Placement at 4% noise. The placement study of main-text Section 7.5 was repeated with 4% instead of 7% observation noise (30 cities, identical seeds). At budget 9 the source NRMSE is 0.378 (GP mutual information), 0.417 (MIST-ID), 0.433 (k-center), 0.434 (all-source

D-optimality), 0.492 (GP variance), and 0.526 (random). GP variance minus MIST-ID: 0.075 $[-0.006, 0.158]$ (0.126 at 7%); full range among the six practical criteria: 0.148 $[0.061, 0.234]$ (0.147 at 7%).

S7. Additional Tables

Table S8. Semantic support prior probes (15 cities): paired MIST-ID-minus-baseline source-NRMSE differences. Positive values favor the baseline. p_{raw} is two-sided Wilcoxon; p_{Holm} applies Holm–Bonferroni over all 12 probes. No probe survives correction.

Probe setting	vs. All-source D-opt			vs. GP mutual information		
	Δ source NRMSE	p_{raw}	p_{Holm}	Δ source NRMSE	p_{raw}	p_{Holm}
Per-budget, budget 5	+0.009	1.00	1.00	-0.080	0.19	1.00
Per-budget, budget 7	+0.081	0.17	1.00	+0.010	0.80	1.00
Per-budget, budget 9	+0.117	0.012	0.15	+0.133	0.018	0.18
Per-budget, budget 11	+0.105	0.012	0.15	+0.130	0.035	0.32
Cross-kernel OSSE, budget 9	+0.037	0.72	1.00	+0.067	0.64	1.00
80-atom dictionary, budget 8	+0.097	0.083	0.67	+0.062	0.11	0.75

Holm–Bonferroni correction over all 12 probes. None survives at $\alpha = 0.05$.

Table S9. Prairie Grass known-origin task at budget 8: release medians with interquartile ranges over 66 releases. The wide, overlapping IQRs are why inference in Section 7.6 of the main text rests on paired within-date contrasts.

Design	Emission error ↓	IQR	Held-out log RMSE ↓	IQR	Releases
Candidate-source D-opt	0.213	[0.133, 0.414]	0.701	[0.491, 1.101]	66
Random	0.380	[0.168, 0.726]	0.726	[0.529, 1.222]	66
Known-source sensitivity	0.517	[0.276, 0.808]	0.801	[0.598, 1.145]	66
Spatial k-center	0.792	[0.458, 0.941]	1.213	[0.681, 1.792]	66
GP variance	0.930	[0.786, 0.967]	1.577	[1.243, 1.943]	66

S8. Additional Figures

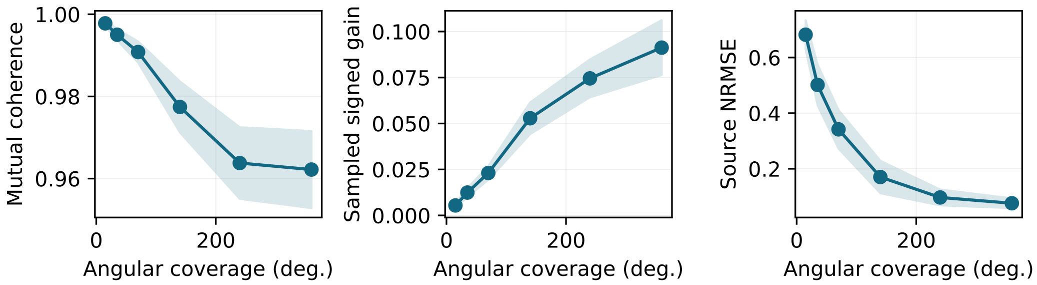

Figure S1. Wind-direction coverage, empirical identifiability, and recovery over 30 cities. Shading is a 95% normal confidence interval over city means. Diverse winds reduce column ambiguity and improve source recovery, but coherence remains high because nearby road atoms are intrinsically similar.

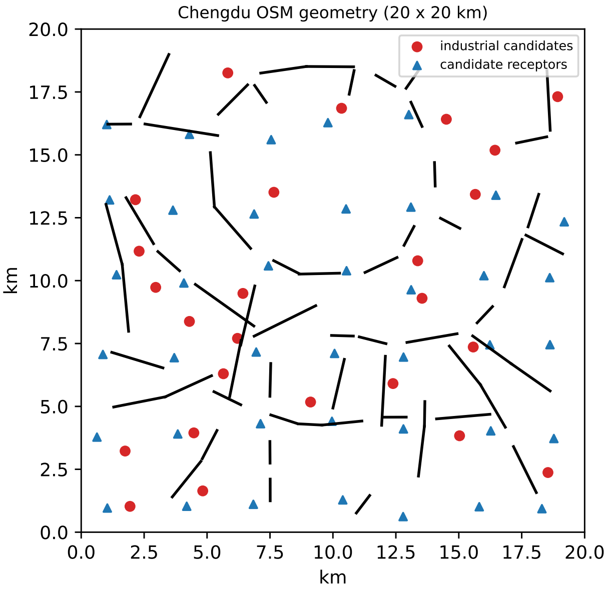

Figure S2. Candidate dictionary built from OpenStreetMap for a 20 × 20 km domain centred on Chengdu: 60 major-road chords (black), 25 industrial land-use clusters (red), and 40 candidate receptor sites (blue).

Reference

1. Mityagin, B.S. The Zero Set of a Real Analytic Function. *Mathematical Notes* **2020**, *107*, 529–530. doi:10.1134/S0001434620030189.
